# Hydroxyapatite-loaded coal gasification slag for synergistic heavy metal adsorption

**DOI:** 10.1016/j.isci.2026.117373

**Published:** 2026-09-07

**Authors:** Xuying Guo, Fanbo Meng, Xiaoyue Zhang, Wei Sun, Yanrong Dong

**Affiliations:** 1 College of Science, College of Mining, Liaoning Technical University, Fuxin 123000, Liaoning, China; 2 College of Mining, Liaoning Technical University, Fuxin 123000, Liaoning, China; 3 College of Civil Engineering, Liaoning Technical University, Fuxin 123000, Liaoning, China; 4 College of Environmental Science and Engineering, Liaoning Technical University, Fuxin 123000, Liaoning, China

**Keywords:** coal gasification slag, nano-HAP, heavy metals, environmental remediation, synergistic adsorption

## Abstract

Lead-zinc mining wastewater contains Pb(II), Cu(II), and Cd(II) that are difficult to remove efficiently. A coal gasification slag-supported nano-hydroxyapatite composite (nHAP-CGS) was prepared via chemical precipitation. The composite achieved removal efficiencies of 97.6%, 94.9%, and 79.8% for Pb(II), Cu(II), and Cd(II), respectively, under optimized conditions (2.5 g/L, pH 4, 120 min, 100 mg/L), outperforming unmodified CGS with a selectivity order of Pb>Cu>Cd. Adsorption followed Langmuir and pseudo-second-order models, indicating chemisorption with intra-particle diffusion. Thermodynamic parameters (*ΔG* < 0, *ΔH* > 0, *ΔS* > 0) confirmed spontaneous endothermic adsorption. BET analysis showed a surface area of 57.65 m^2^/g for nHAP-CGS, significantly higher than raw CGS. Coexisting-ion tests and leaching experiments confirmed selectivity and environmental safety. This work provides a basis for coal gasification slag valorization and heavy metal remediation.

## Highlights

• A novel adsorbent was prepared by loading nano-hydroxyapatite on coal gasification slag
• The composite showed excellent synergistic adsorption for Pb(II), Cu(II), and Cd(II)
• Competitive adsorption behavior among three heavy metals was systematically investigated
• The material has great potential for treating heavy metal wastewater from mining areas

## Graphical abstract

**Figure:**
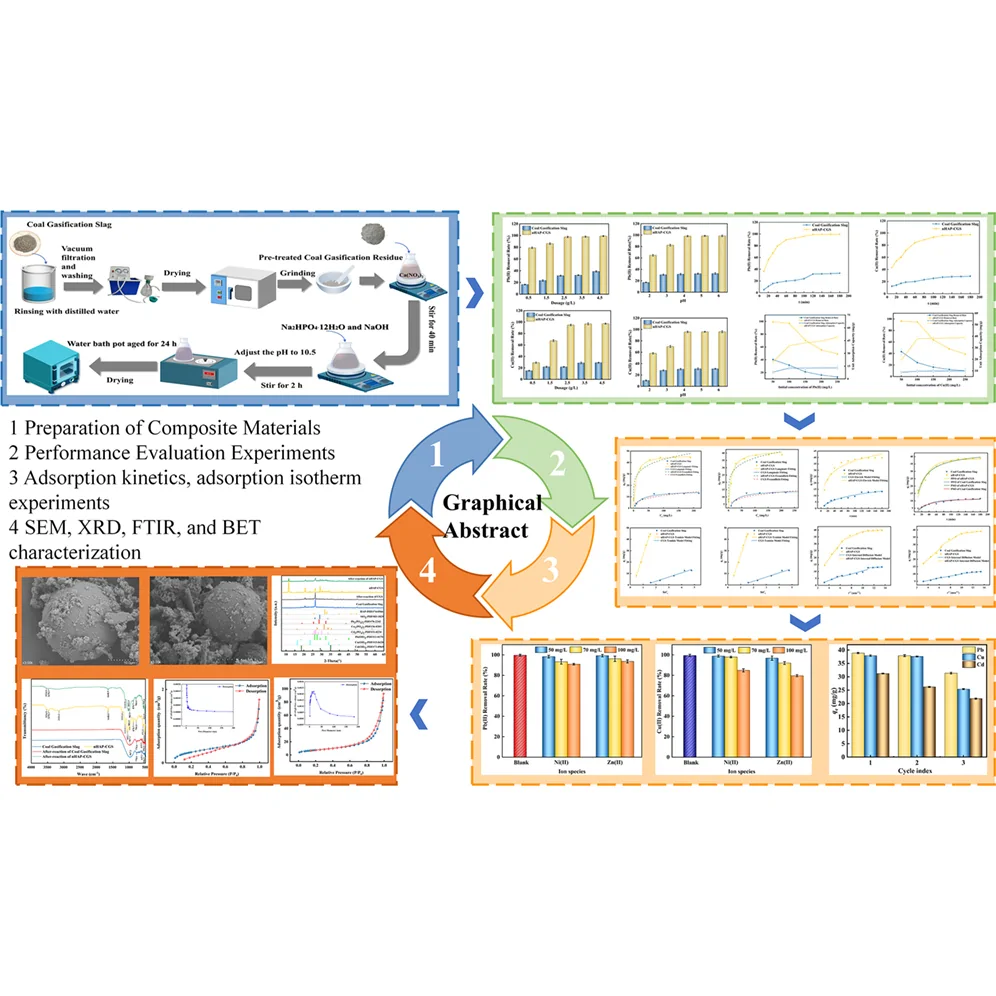

## Teaser

Environmental resource; Environmental science; Pollution

## Introduction

China has been recognized as a major producer and smelter of lead-zinc ore, and extensive mining has been accompanied by the discharge of large quantities of heavy-metal-laden wastewater from lead-zinc mining areas.^1^^,^^2^^,^^3^ This wastewater is typically acidic, enriched in Pb(II) and Zn(II), and frequently contains additional toxic metals such as Cu(II) and Cd(II), posing serious threats to ecosystems and human health.^4^^,^^5^^,^^6^ Although Zn(II) can often be removed efficiently by conventional lime neutralization and coagulation-precipitation,^7^ Pb(II), Cu(II), and Cd(II) exhibit stronger bioaccumulation and irreversible neurotoxicity and are therefore regarded as priority pollutants requiring urgent control.^5^^,^^6^^,^^8^ Treatment of lead-zinc mine wastewater is currently dominated by chemical precipitation,^9^ ion exchange,^10^ biological processes,^11^ and adsorption.^12^ Each method has its advantages and limitations. The chemical precipitation method is simple and cost-effective, but it generates a large amount of harmful sludge, which often fails to meet heavy metal emission standards. Ion exchange has high selectivity and good regenerability, but its application is limited by high material costs and sensitivity to suspended solids. Biological processes are environmentally friendly, but their dynamics are slow, they are sensitive to toxic effects, and microbial cultivation is difficult. In contrast, the characteristics of adsorption are simple operation, wide pH tolerance, and great potential for adsorbent regeneration. However, its practical application largely depends on the development of low-cost, high-capacity, and reusable adsorbents. Thus, low-cost adsorbents with high removal rates are urgently needed in mine environmental remediation.

Coal gasification slag (CGS), generated as a solid by-product during coal gasification, has become a major barrier to the sustainable development of the coal industry.^13^^,^^14^^,^^15^ At present, CGS is mainly used in low value-added applications (e.g., construction materials and roadbed fill),^16^^,^^17^^,^^18^ and technologies for its high-value utilization remain immature. Consequently, resource-oriented utilization of CGS is a pressing challenge. Owing to its high contents of Si, Al, and Ca and its porous structure, CGS has been explored as an adsorbent for environmental remediation.^19^^,^^20^ Yet, when used alone, CGS suffers from low specific surface area, few active sites, and limited adsorption capacity, restricting practical application.^21^ To enhance performance, approaches such as alkali modification, surfactant treatment, and loading of functional phases have been adopted. Nonetheless, alkali and membrane-type modifications tend to be costly, potentially cause secondary pollution, or deliver only single functionality,^21^^,^^22^ whereas utilization of CGS as a carrier for active components can provide low-cost, multifunctional, and efficient adsorption through synergistic effects and is considered a more promising route.^23^

Nano-hydroxyapatite (nHAP) has been widely investigated as a heavy-metal sorbent because of its abundant surface functional groups and strong affinity toward metal ions.^24^^,^^25^^,^^26^ Phosphate and hydroxyl groups on nHAP surfaces can immobilize heavy metals via ion-exchange and surface complexation.^27^ Recent studies have demonstrated the excellent performance of nHAP-based materials for heavy metal removal. For instance, nHAP synthesized from clam shells achieved adsorption capacities of 265, 64, and 55 mg/g for Pb(II), Cd(II), and Cu(II), respectively.^28^ Carbonated nHAP derived from pig bone waste exhibited exceptionally high adsorptivity for multiple heavy metals, with strontium adsorption being 250 times higher than that of normal bone.^29^ These examples highlight the strong potential of nHAP as an effective adsorbent, while also revealing that its practical application often requires immobilization on suitable carriers to overcome the challenges of nanoparticle agglomeration and poor mechanical stability.^30^ To address this, nHAP has been immobilized on porous supports to improve dispersion and mechanical stability. For example, nHAP-loaded montmorillonite has been applied to Cd(II)-containing wastewater with markedly increased adsorption capacity,^31^ and nHAP/graphene oxide composites have exhibited enhanced synergistic adsorption of multiple heavy metals.^32^ These findings indicate that loading nano-HAP onto structurally robust carriers effectively mitigates agglomeration and mechanical weakness while improving adsorption performance. Recent research has further promoted the development of efficient adsorbents for removing heavy metals using various materials. For example, Xie et al.^33^ constructed a carboxylated graphene oxide/MOF-808 composite material that follows Langmuir and second-order kinetic models for Pb(II), Cd(II), and Cu(II) adsorption. Li et al.^34^ synthesized bagasse-derived carboxymethyl cellulose aerogel to remove Pb(II) and other pollutants, and used EDTA as an effective desorption agent for regeneration. The same group also reported that cellulose poly (acrylic acid) hydrogel derived from bagasse removed Pb(II), Cu(II), and Cd(II) through cation exchange and electrostatic interaction.^35^ These studies collectively emphasize the importance of adsorption mechanisms and regeneration experiments. With its dense framework dominated by inert aluminosilicate minerals and glassy phases, CGS can act as an ideal mineral skeleton for hosting nanomaterials. Therefore, CGS is selected here as a carrier for nano-HAP to construct a composite adsorbent, with the aim of enabling high-value utilization of CGS and providing a viable option for lead-zinc mine wastewater remediation.

In this work, we fabricated a coal gasification slag-loaded nano hydroxyapatite composite material (nHAP-CGS) using the chemical precipitation method. The specific objectives of this study are: (1) to characterize the structure and surface properties of nHAP-CGS using SEM, XRD, FTIR, and BET techniques; (2) Evaluate its adsorption performance for Pb(II), Cu(II), and Cd(II) under various conditions (dosage, pH, reaction time, and initial concentration); (3) Elucidate the synergistic adsorption mechanism through isotherm, kinetic, and thermodynamic analysis; (4) Evaluate the reusability and environmental safety of composite materials through cyclic regeneration and leaching toxicity tests. This work aims to provide a theoretical basis and technical reference for the resource utilization of coal gasification slag and the treatment of lead-zinc mine wastewater.

## Results and analysis

### Performance evaluation

#### Effect of adsorbent dosage

**Figure 1:**
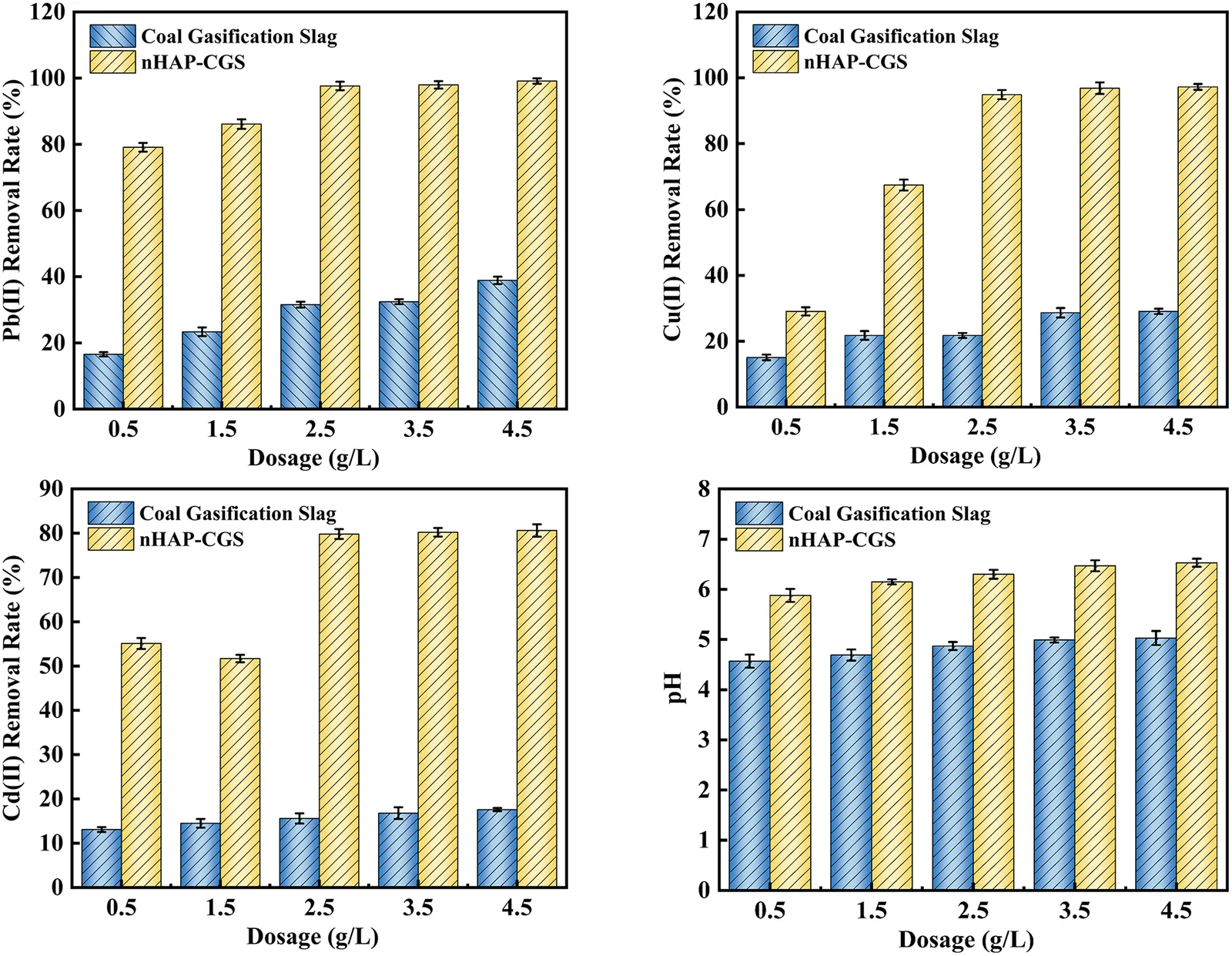
The effects of adsorbent dosage on removal of Pb(II), Cu(II), Cd(II), and pH (all data are represented as mean ± SD)

The effect of adsorbent dosage on the removal of Pb(II), Cu(II), and Cd(II) and on solution pH is illustrated in Figure 1. With increasing dosage, removal efficiencies of Pb(II), Cu(II), and Cd(II) by nHAP-CGS are consistently enhanced. When the CGS dosage is increased from 0.5 g/L to 4.5 g/L, removal efficiencies of Pb(II), Cu(II), and Cd(II) are only increased from 16.56% to 38.88%, 15.10%–29.10%, and 13.10%–17.60%, respectively, accompanied by a pH increase from 4.57 to 5.03. Under the same dosage range, removal efficiencies on nHAP-CGS are increased from 79.10% to 99.12% for Pb(II), from 29.08% to 97.24% for Cu(II), and from 55.10% to 80.60% for Cd(II), while pH increases from 5.88 to 6.53. Thus, the adsorption capacity of CGS is markedly improved after nHAP loading, especially for Pb(II) and Cu(II). At lower dosages, the increase in nHAP-CGS dosage leads to a pronounced increase in available surface area and adsorption sites, so adsorption of Pb(II), Cu(II), and Cd(II) is strongly promoted. Once the dosage exceeds 2.5 g/L, the incremental gains in Pb(II) and Cu(II) removal become less significant, and the Cd(II) removal curve also levels off beyond 3.5 g/L. This behavior suggests that, at higher dosages, the residual metal concentration in solution becomes low, the mass-transfer driving force is weakened, and further improvement in removal rate is limited.^36^ The rise in pH induced by nHAP-CGS favors metal hydroxide formation and strengthens surface complexation, thereby contributing to enhanced removal, whereas the moderate change in pH indicates that nHAP-CGS provides a limited buffering effect and does not cause drastic pH shifts.^37^ At a dosage of 2.5 g/L, removal efficiencies for Pb(II), Cu(II), and Cd(II) reach 97.64%, 94.88%, and 79.80%, respectively, corresponding to adsorption capacities of 39.06, 37.95, and 31.92 mg/g. Compared with raw CGS (Figures 1A–1C), nHAP-CGS exhibits significantly higher adsorption capacities under all tested dosages, confirming that nHAP loading greatly enhances the adsorption performance. Consequently, 2.5 g/L is selected as the optimal dosage in subsequent tests, striking a balance between high removal rate and economic adsorbent usage.

#### Effect of initial solution pH

**Figure 2:**
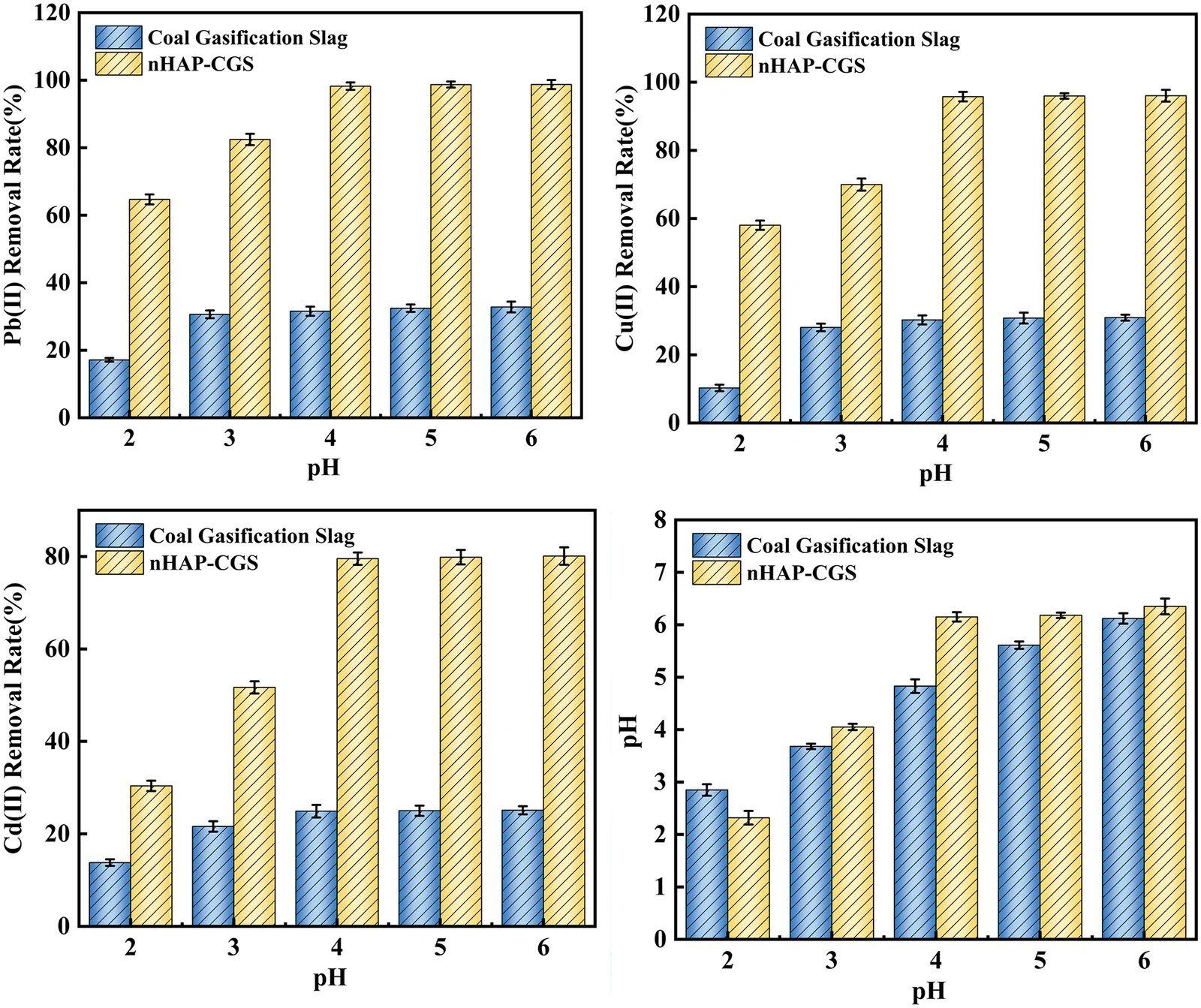
The effects of initial solution pH on the removal of Pb(II), Cu(II), and Cd(II) (all data are represented as mean ± SD)

The influence of initial solution pH on metal removal is shown in Figure 2. For both CGS and nHAP-CGS, removal efficiencies of Pb(II), Cu(II), and Cd(II) increase with increasing initial pH. When the initial pH is raised from 2 to 4, the removal efficiencies of Pb(II), Cu(II), and Cd(II) by nHAP-CGS are enhanced from 64.71%, 58.04%, and 30.40%–98.26%, 95.78%, and 79.53%, respectively, and the final pH values after reaction stabilize at approximately 6.15, corresponding to adsorption capacities of 39.30, 38.31, and 31.81 mg/g at pH 4. In contrast, raw CGS achieved only 38.88%, 29.10%, and 17.60% removal at pH 4 (Figure 2), further demonstrating the superior adsorption capacity of nHAP-CGS. Under strong acidity (pH = 2–3), high H^+^ concentrations result in intense competition with Pb(II), Cu(II), and Cd(II) for surface active sites, and protonation of surface groups generates positively charged species (e.g., ≡CaOH_2_^+^), giving rise to electrostatic repulsion toward metal cations and suppressing adsorption.^38^ As the pH value increases above the isoelectric point (usually pH 4–5), the surfaces of nHAP and CGS-derived materials become increasingly negative, which enhances the electrostatic attraction to Pb(II), Cu(II), and Cd(II), and explains the observed improvement in removal efficiency at higher pH.^39^ At pH 4–6, the H^+^ concentration is reduced, competition between H^+^ and metal ions is weakened, and more surface sites on nHAP-CGS remain available for binding Pb(II), Cu(II), and Cd(II). As a result, removal efficiencies increase and then become stable at initial pH ≥ 4. Over this pH range, nHAP-CGS exhibits robust adsorption performance. Considering typical pH levels of lead-zinc mine wastewater, treatment feasibility, and to avoid the potential interference of metal hydroxide precipitation (which may occur at pH > 5), an initial pH of 4 was adopted in subsequent experiments. Notably, even at pH 6 (Figure 2), the removal trend remains gradual without an abrupt increase, confirming that precipitation is not the dominant mechanism.

#### Effect of reaction time

**Figure 3:**
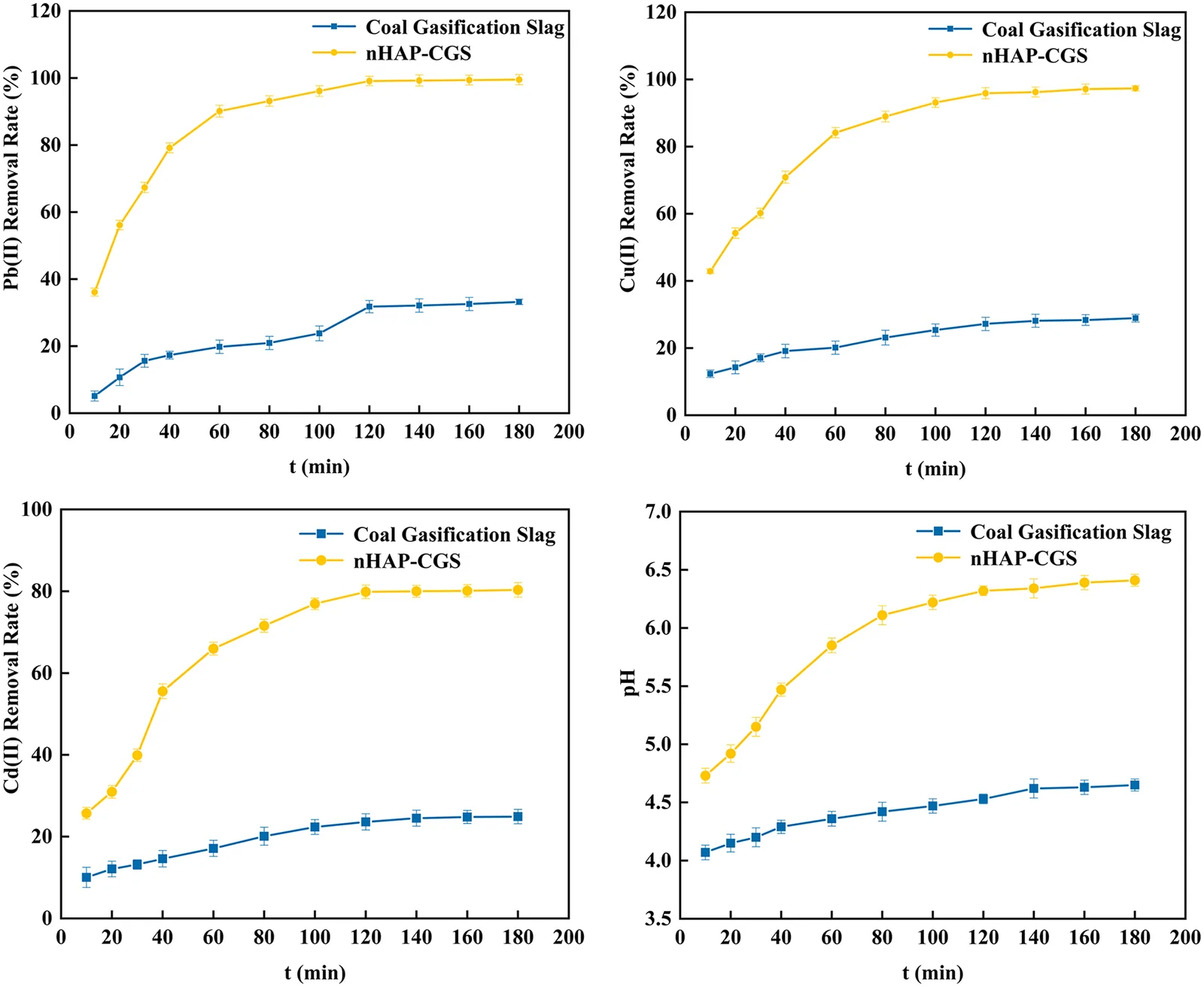
Effect of reaction time on the removal of Pb(II), Cu(II), and Cd(II) (all data are represented as mean ± SD)

The time-dependent performance of CGS and nHAP-CGS is summarized in Figure 3. For Pb(II), removal by nHAP-CGS increases rapidly from 36.12% to 99.12% within 10–120 min and reaches 99.52% at 180 min, whereas removal by CGS increases only from 5.13% to 33.21%. For Cu(II), the removal rate of nHAP-CGS rises from 42.89% to 95.89% in 10–120 min and further to 97.34% at 180 min, while that of CGS increases from 12.36% to 28.90%. For Cd(II), nHAP-CGS exhibits an increase from 25.70% to 79.84% in 10–120 min and to 80.34% at 180 min, compared with a rise from 10.05% to 24.90% for CGS. For comparison, raw CGS achieved only 33.21%, 28.90%, and 24.90% removal for Pb(II), Cu(II), and Cd(II) at 120 min (Figure 3), confirming that nHAP loading significantly accelerates the adsorption rate and increases the equilibrium capacity. The evolution of pH also reveals a clear distinction: in the nHAP-CGS system, pH increases from 4.73 to 6.41 by 180 min, while in the CGS system it increases only from 4.07 to 4.65. This indicates stronger buffering capacity and alkalinity release by nHAP-CGS. At the initial stage of adsorption, abundant high-energy sites and large surface area on nHAP-CGS enable rapid complexation and precipitation with metal ions, accompanied by OH^−^ release, which elevates pH.^40^ As time proceeds, surface sites approach saturation and solution metal concentrations decrease, so the mass-transfer driving force diminishes, and the adsorption rate decreases. The current sampling scheme (10–180 min) captured the overall kinetic curve and confirmed that equilibrium was reached within 120 min. Although more intensive sampling in the first 10 min would further improve the estimation of initial adsorption rate, the existing data have provided an excellent fit for second-order and intraparticle diffusion models, supporting the proposed adsorption mechanism. To ensure adequate equilibration in subsequent experiments, a contact time of 120 min is chosen.

#### Effect of initial concentration

**Figure 4:**
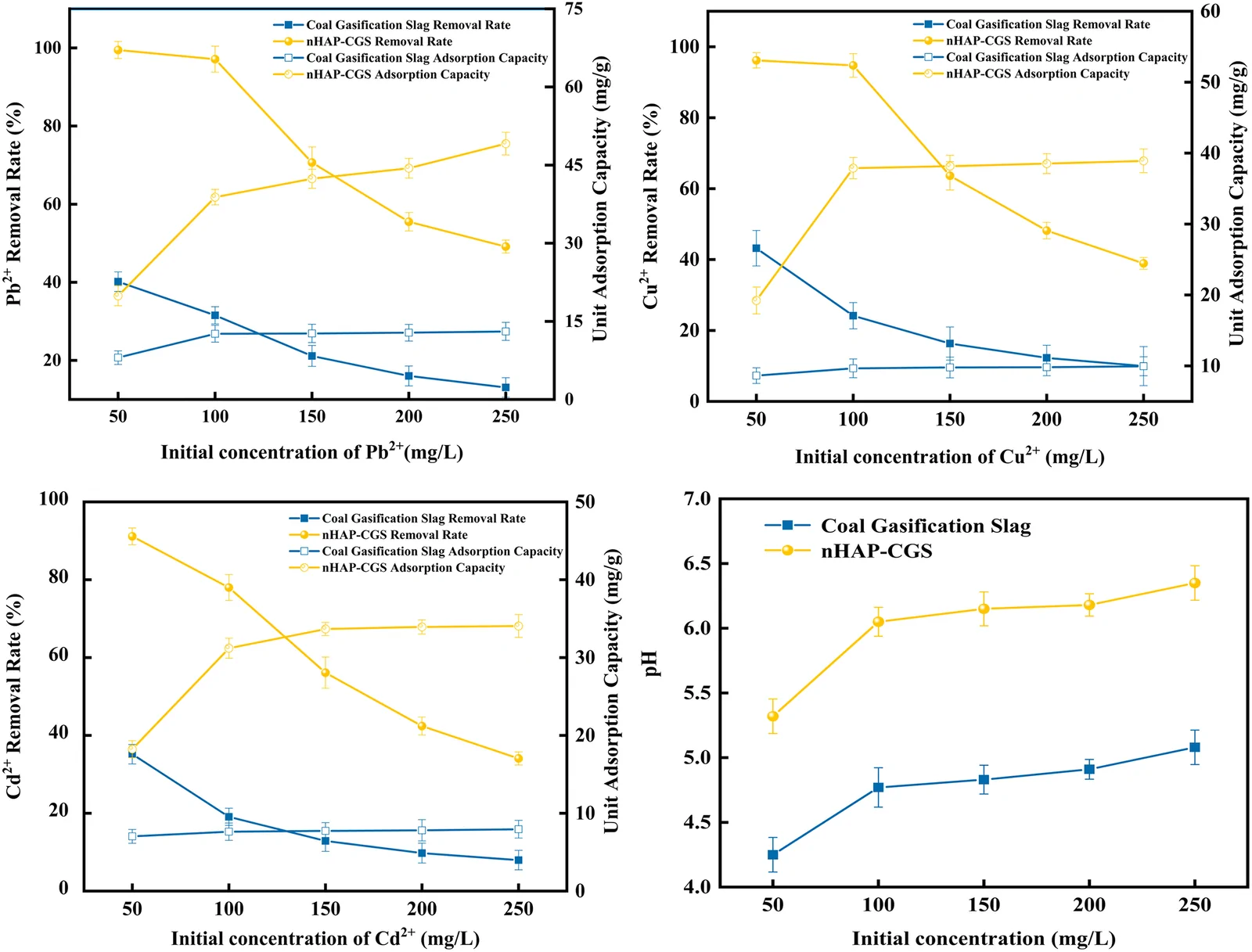
Effect of initial concentration on removal of Pb(II), Cu(II), and Cd(II) (all data are represented as mean ± SD)

The effect of initial metal concentration is depicted in Figure 4. For Pb(II), an increase in initial concentration from 50 mg/L to 250 mg/L causes the removal rate of nHAP-CGS to decrease from 99.46% to 49.12%, while the corresponding adsorption capacity increases from 19.892 mg/g to 49.12 mg/g. For Cu(II), removal decreases from 96.16% to 38.92%, while adsorption capacity increases from 19.232 mg/g to 38.92 mg/g. For Cd(II), removal decreases from 91.15% to 34.06%, with adsorption capacity increasing from 18.23 mg/g to 34.06 mg/g. By contrast, CGS exhibits much lower removal efficiencies and adsorption capacities for all three metals, and these values vary only slightly with concentration, indicating a limited number of effective adsorption sites. As initial concentration increases, the pH of the CGS system rises from 4.25 to 5.08, while that of the nHAP-CGS system rises from 5.32 to 6.35; in both cases, the pH remains within a weakly acidic to near-neutral range favorable for metal uptake. At low initial concentrations, numerous vacant sites on nHAP-CGS allow extensive adsorption and ion-exchange with Pb(II), Cu(II), and Cd(II), yielding high removal efficiencies. As concentration increases, these sites become progressively occupied and approach saturation, resulting in decreasing removal efficiencies. However, the increased concentration of metal ions enhances the mass-transfer driving force toward the adsorbent surface, so the total metal uptake per unit mass continues to increase until saturation is approached.^41^ Consequently, adsorption capacities increase with concentration and then level off. To ensure effective removal of all three metals, an initial concentration of 100 mg/L is selected for subsequent studies.

### Analysis of adsorption isotherms of Pb(II), Cu(II), and Cd(II)

**Figure 5:**
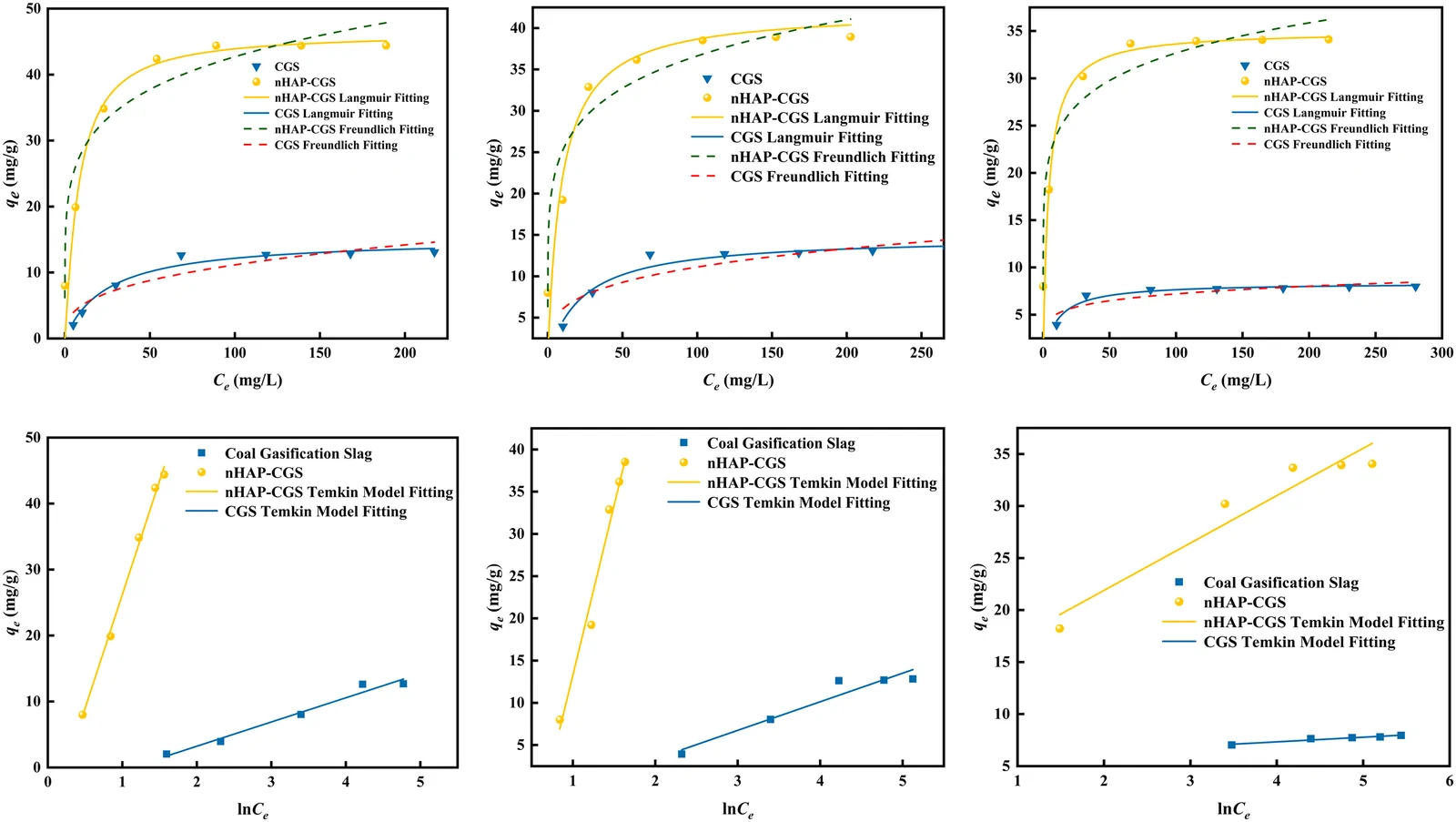
Isotherm model fitting for Pb(II), Cu(II), and Cd(II) adsorption by coal gasification slag and nHAP-CGS

The equilibrium behavior of Pb(II), Cu(II), and Cd(II) adsorption at the solid-liquid interface was characterized by adsorption isotherms. The Langmuir, Freundlich, and Temkin isotherm models were employed to fit the adsorption isotherms of these metal ions on nHAP-CGS, and the corresponding results are shown in Figure 5 and Table 1.

**Table 1:** Adsorption isotherm parameters of Pb(II), Cu(II), and Cd(II) by CGS and nHAP-CGS

Model	Parameters	Pb(II)		Cu(II)		Cd(II)	
		CGS	nHAP-CGS	CGS	nHAP-CGS	CGS	nHAP-CGS
Langmuir	*q*_*m*_	15.223	46.3080	14.7982	42.1919	8.3657	35.0148
	*K*_*L*_	0.0397	0.0884	0.0441	0.1094	0.1048	0.2431
	*R*^*2*^	0.9709	0.98631	0.9479	0.9149	0.9492	0.9439
Freundlich	*n*	2.8923	18.7795	3.8016	6.1656	6.4973	7.436
	*K*_*F*_	2.2719	5.6035	3.3096	17.3615	3.5458	17.5908
	*R*^*2*^	0.8482	0.92179	0.7756	0.88543	0.7158	0.8787
Temkin	*K*_*T*_	0.3260	0.7877	0.3648	0.5120	0.5644	16.5836
	*B*	3.6708	34.4376	3.3849	40.1749	0.4457	4.5531
	*R*^*2*^	0.9661	0.9944	0.9050	0.9724	0.9335	0.9063

A comparison of the maximum adsorption capacities (*q*_*m*_) obtained from the Langmuir model reveals that nHAP-CGS exhibits significantly higher *q*_*m*_ values for Pb(II) (46.31 mg/g), Cu(II) (42.19 mg/g), and Cd(II) (35.01 mg/g) than raw CGS (15.22, 14.80, and 8.37 mg/g, respectively). This confirms that loading nano-hydroxyapatite onto coal gasification slag greatly enhances the adsorption capacity. Among the three metal ions, the *q*_*m*_ follows the order Pb(II) > Cu(II) > Cd(II) for both adsorbents. From the fitted curves and experimental data, it is observed that the Langmuir model on nHAP-CGS provides consistently higher coefficients of determination (*R*^*2*^ = 0.98631, 0.9149, and 0.9439) than the other two models. For Pb(II) and Cd(II), the *R*^*2*^ values of the Langmuir model are all above 0.94, and an *R*^2^ value as high as 0.9863 is obtained for Pb(II) on nHAP-CGS. These results suggest that the Langmuir model can effectively describe the overall adsorption behavior, implying that adsorption predominantly occurs on energetically homogeneous sites and forms a monolayer coverage. The Temkin model assumes that the heat of adsorption decreases linearly with increasing surface coverage due to adsorbateadsorbent interactions. The excellent fit of the Temkin model to Pb(II) adsorption on nHAP-CGS (*R*^*2*^ = 0.9944) therefore indicates that the adsorption heat is not constant but gradually decreases as more Pb(II) ions are adsorbed. This implies that Pb(II) preferentially occupies high-energy active sites (e.g., phosphate and hydroxyl groups on nHAP) during the initial adsorption stage, followed by binding to lower-energy sites as the surface becomes saturated. The decreasing trend of adsorption heat may also reflect lateral repulsive interactions among adsorbed Pb(II) species or the inherent surface heterogeneity of nHAP-CGS. For Cu(II) (*R*^*2*^ = 0.9724) and Cd(II) (*R*^*2*^ = 0.9063), the slightly lower *R*^2^ values suggest that their adsorption heat distributions deviate more from the linear Temkin assumption, likely due to weaker or more complex adsorbate-adsorbent interactions. In contrast, the Freundlich model gives relatively lower *R*^*2*^ values (0.92179, 0.88543, and 0.8787), although it is commonly used to describe multilayer adsorption on heterogeneous surfaces. The poorer fit indicates that surface heterogeneity is not the dominant mechanism. Taken together, these observations indicate that the adsorption of Pb(II), Cu(II), and Cd(II) on both adsorbents is better represented by the Langmuir model and is mainly governed by monolayer adsorption.

### Analysis of adsorption kinetics of Pb(II), Cu(II), and Cd(II)

**Figure 6:**
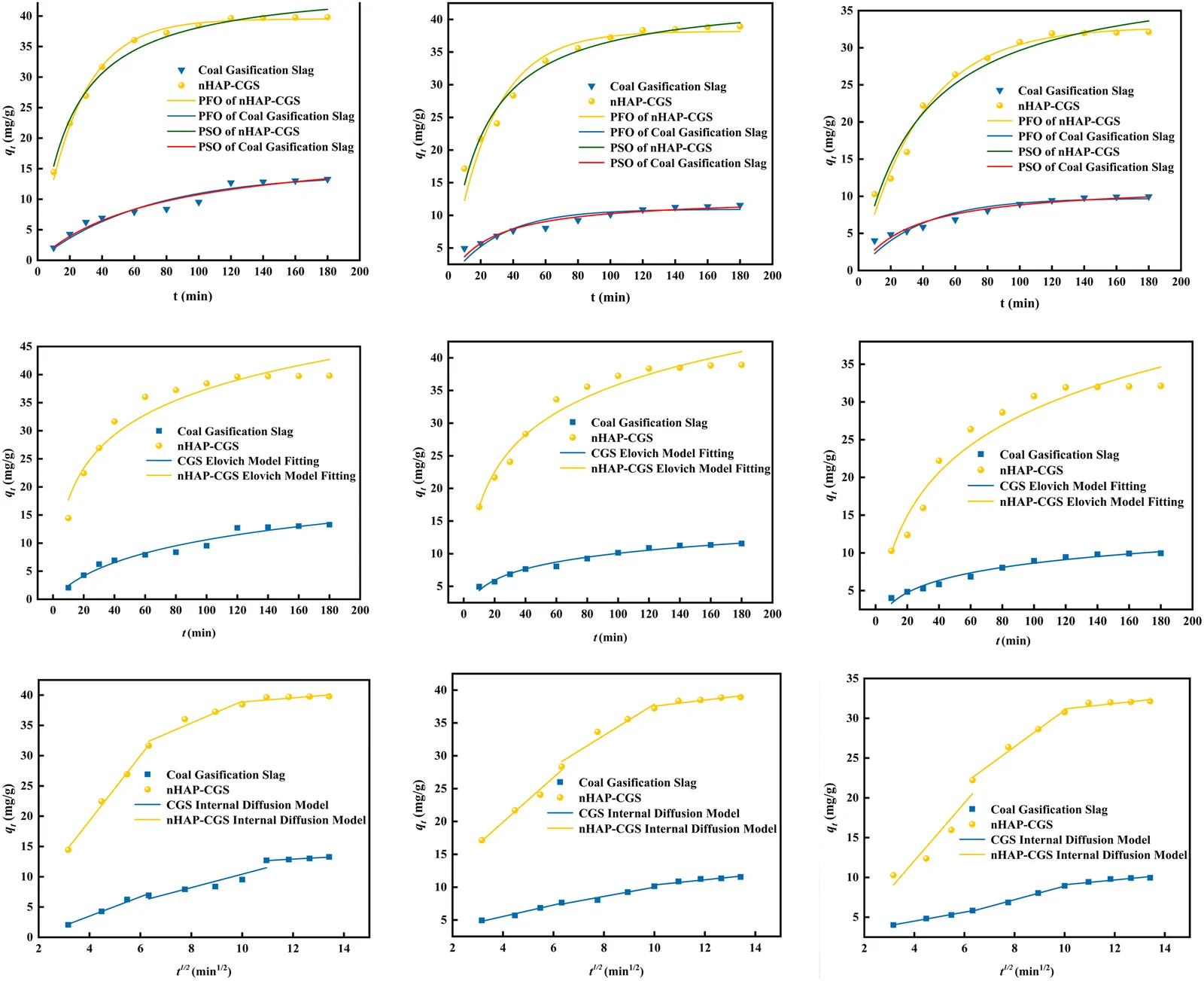
Kinetic model fitting diagram for Pb(II), Cu(II), and Cd(II) Adsorption by coal gasification residue and nHAP-CGS

The adsorption kinetics of Pb(II), Cu(II), and Cd(II) on coal gasification slag (CGS) and nHAP-CGS were analyzed to clarify the adsorption characteristics and rate-controlling mechanisms. The pseudo-first-order (PFO), pseudo-second-order (PSO), and intraparticle diffusion (IPD) models were used to fit the kinetic data for nHAP-CGS, and the resulting parameters and curves are presented in Figure 6 and Table 2. On the basis of the *R*^*2*^ values, distinct differences in kinetic descriptions are evident. As listed in Table 2, the PSO model for nHAP-CGS yields higher *R*^2^ values for Pb(II), Cu(II), and Cd(II) (0.9865, 0.9743, and 0.9787, respectively) than the PFO model (0.9643, 0.9360 and 0.9587). The corresponding equilibrium capacities obtained from the PSO model (39.235, 38.183, and 32.795 mg/g) are closer to the experimentally measured values, indicating that the PSO model is more appropriate for describing the adsorption of Pb(II), Cu(II), and Cd(II) on nHAP-CGS. This finding suggests that the overall process is mainly controlled by chemisorption. During adsorption, pollutants are first bound at reactive sites on the external surface of the adsorbent until saturation, and subsequent diffusion into the internal pore network then contributes to further uptake.

**Table 2:** Adsorption kinetics parameters of Pb (II), Cu (II), and Cd (II) by CGS and nHAP-CGS

Model	Parameters	Pb(II)		Cu(II)		Cd(II)	
		CGS	nHAP-CGS	CGS	nHAP-CGS	CGS	nHAP-CGS
Pre-level 1	*k*_1_	0.0148	0.0407	0.0325	0.0389	0.1611	0.0264
	*q*_e_	4.14	45.556	3.932	43.8601	9.748	40.3984
	*R*^*2*^	0.9426	0.9643	0.8523	0.9360	0.8804	0.9587
Pre-level 2	*k*_*2*_	0.0067	0.0011	0.0031	0.0012	0.0026	0.0007
	*q*_e_	9.0794	39.235	3.8006	38.183	2.6924	32.795
	*R*^*2*^	0.9534	0.9865	0.9336	0.9743	0.9327	0.9787
Internal diffusion	*k*_p1_	1.5958	5.3709	0.8728	3.4054	0.5629	3.6347
	*C*_1_	−2.8769	−2.2235	2.0460	6.2732	2.2599	−2.4481
	*R*^*2*^	0.9828	0.9944	0.9698	0.9756	0.9926	0.8424
	*k*_p2_	1.1103	1.7962	0.6995	2.3769	0.8552	2.2962
	*C*_2_	−0.6711	21.0267	2.9989	14.0901	0.3595	8.0489
	*R*^*2*^	0.7707	0.9216	0.9183	0.9216	0.9934	0.9820
	*k*_p3_	0.2299	0.3412	0.3921	0.4608	0.2999	0.3421
	*C*_3_	10.1584	35.456	6.4206	32.9527	6.0817	27.7513
	*R*^*2*^	0.9516	0.51922	0.9183	0.7979	0.8597	0.5508
Elovich	*α*	0.3035	5.4339	1.0781	5.4307	0.6537	1.7286
	*β*	0.1731	0.1098	0.3687	0.1157	0.3744	0.1003
	*R*^*2*^	0.9593	0.9326	0.9778	0.9675	0.9670	0.9465

The PSO rate constants *k*_*2*_ (1.1 × 10^−3^, 1.2 × 10^−3^ and 0.7 × 10^−3^) are markedly lower than the corresponding PFO rate constants *k*_*1*_ (0.0407, 0.0389 and 0.0264), indicating that, although the intrinsic chemisorption rate of nHAP-CGS is relatively slow, the affinity of surface binding sites for Pb(II), Cu(II), and Cd(II) is high and promotes efficient adsorption. The *R*^*2*^ values of the PSO model for nHAP-CGS are comparable to those obtained for CGS (0.9534, 0.9336 and 0.9327); however, the *k*_2_ values for nHAP-CGS are smaller than those for CGS (0.67 × 10^−2^, 0.31 × 10^−2^, and 0.26 × 10^−2^), whereas the equilibrium capacities *q*_*e*_ (39.235, 38.183 and 32.795 mg/g) exceed those of CGS (9.0794, 3.8006 and 2.6924 mg/g). It can therefore be inferred that adsorption on nHAP-CGS requires a longer contact time to establish equilibrium but provides a significantly higher pollutant removal capacity than CGS. For raw CGS, although the pseudo-second-order model provides good fits (*R*^*2*^ > 0.93), this alone is not definitive evidence of chemisorption, as the model can also describe physical adsorption processes that are limited by site availability.

To elucidate the diffusion mechanism in more detail, the IPD model was further applied to the kinetic data. The IPD plots for all three ions exhibit obvious multi-linear trends, indicating that the adsorption process can be divided into three characteristic stages: external diffusion, intraparticle diffusion, and final equilibrium. At the initial stage, a large number of vacant sites on the nHAP-CGS surface lead to a rapid increase in *q*_*t*_, reflecting a fast uptake step dominated by film diffusion and micropore diffusion from the bulk solution to the outer surface. As adsorption proceeds, surface sites become progressively occupied; the increase in q_t_ slows, and solute species that are already adsorbed migrate into the internal pore structure, giving rise to a pore-filling process. In the final stage, the number of available sites approaches saturation, *q*_*t*_ becomes nearly constant, the effective diffusion rate decreases sharply and a quasi-equilibrium state is attained. Because none of the IPD fitting lines pass through the origin, the adsorption of Pb(II), Cu(II), and Cd(II) on nHAP-CGS is confirmed to be controlled by the combined effects of surface diffusion and intraparticle diffusion, rather than by a single rate-limiting step.

The Elovich model is commonly used to describe chemical adsorption on heterogeneous surfaces and fits the kinetic data well, with *R*^*2*^ values ranging from 0.9326 to 0.9675 for nHAP-CGS. The initial adsorption rate constant (*α*) of nHAP CGS for Pb(II) is similar to that of Cu(II), but much higher than that of Cd(II), indicating that Pb(II) and Cu(II) have faster adsorption rates in the initial stage. This is consistent with the observed high affinity of nHAP for Pb(II) and Cu(II) in isotherm experiments. The beta values related to surface coverage are comparable for the three ions, indicating that once surface active sites are occupied, further adsorption becomes increasingly difficult. For CGS, the alpha value is significantly lower than that of nHAP-CGS, and the beta value is higher than that of nHAP-CGS, further confirming the superior adsorption kinetics of the composite material.

### Analysis of adsorption thermodynamics of Pb(II), Cu(II), and Cd(II)

**Figure 7:**
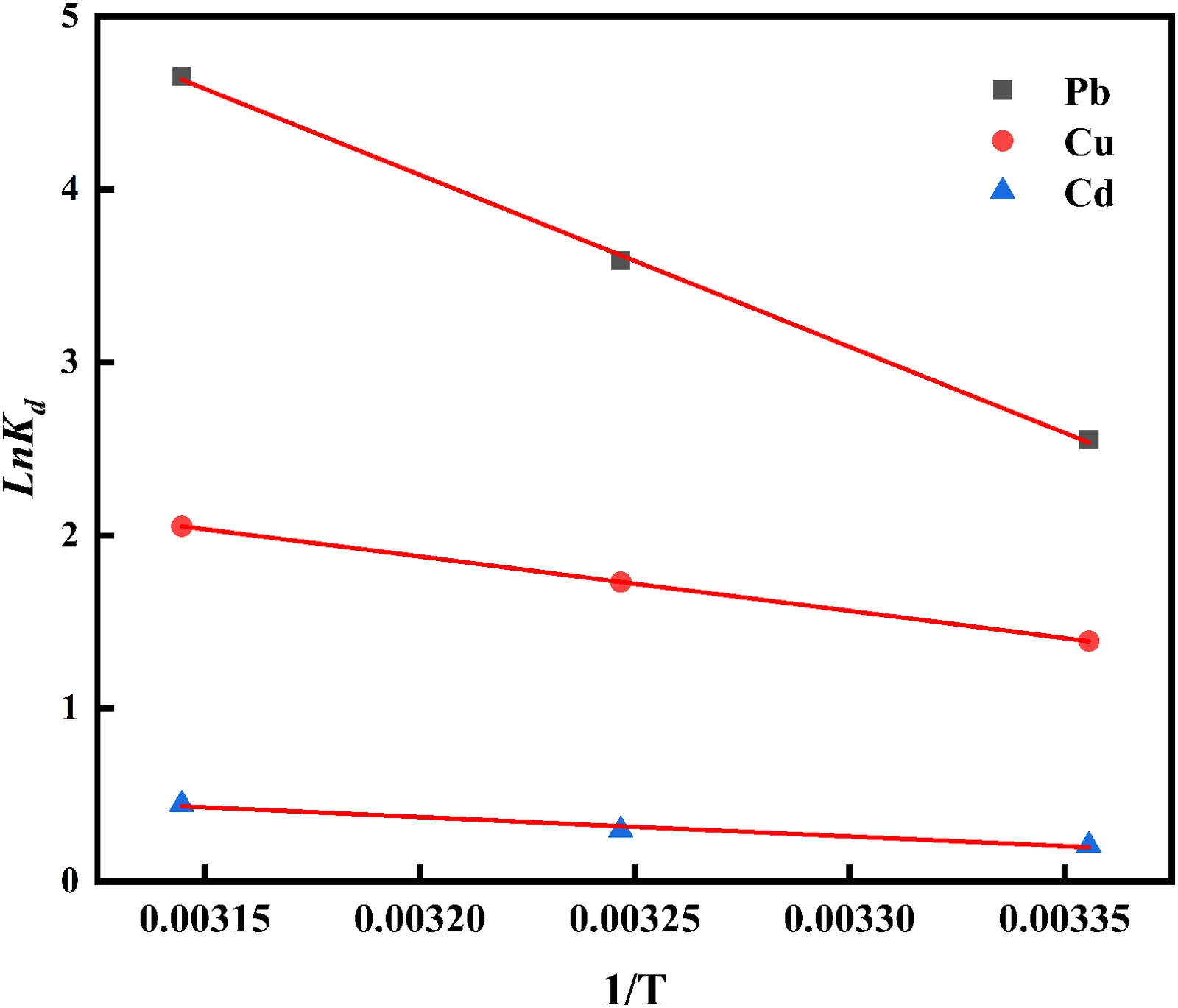
Thermodynamic model fitting diagram for nHAP-CGS adsorption of Pb(II), Cu(II), and Cd(II)

Thermodynamic parameters for the adsorption of Pb(II), Cu(II), and Cd(II) on nHAP-CGS were evaluated at 298.15, 308.15, and 318.15 K with an initial pH of 4 and an initial metal concentration of 100 mg/L. For each metal, the distribution coefficient *K*_*d*_ was calculated and used to construct ln*K*_*d*_^−1^/*T* plots, which were then fitted linearly in Figure 7. The slope and intercept of each linear fit were used to determine the enthalpy change (*ΔH*) and entropy change (*ΔS*), and the resulting parameters are summarized in Table 3.

**Table 3:** Adsorption thermodynamic parameters of nHAP-CGS for the adsorption of Pb^2+^, Cu^2+^, and Cd^2+^

	*ΔG*(kJ/mol)			*ΔH*(kJ/mol)	*ΔS*(J/(mol·K))
	298 K	308 K	318 K		
Pb (II)	−6.3251	−6.6262	−12.3009	82.6723	298.5171
Cu (II)	−3.4439	−4.4307	−5.4296	26.1419	99.2751
Cd (II)	−0.5182	−0.7593	−1.1802	9.3162	32.9074

In the temperature range of 298–318 K, the adsorption of Pb(II), Cu(II), and Cd(II) on nHAP-CGS occurs spontaneously, as evidenced by negative *ΔG* values that become more negative with increasing temperature (Pb: −6.33 to −12.30 kJ/mol; Cu: −3.44 to −5.43 kJ/mol; Cd: −0.52 to −1.18 kJ/mol). This behavior indicates that higher temperatures enhance adsorption. The magnitude of *ΔG* follows the order |*ΔG*_*-Pb*_|>|*ΔG*_*-Cu*_|>|*ΔG*_*-Cd*_|, which is consistent with the order of adsorption capacities and affinities and indicates that both the driving force and the uptake capacity of nHAP-CGS are greatest for Pb(II). The positive *ΔH* values (Pb: 82.67 kJ/mol; Cu: 26.14 kJ/mol; Cd: 9.32 kJ/mol) confirm that adsorption is endothermic. The relatively high *ΔH* values for Pb(II) and Cu(II) point to chemisorption as the dominant mechanism, whereas the smaller *ΔH* value for Cd(II) suggests a larger contribution from physisorption.

The positive *ΔS* values for all three ions imply that dehydration of hydrated metal ions releases a substantial number of water molecules, increasing the randomness of the system; the resulting entropy gain thus constitutes a key driving force for adsorption. Overall, the adsorption of Pb(II), Cu(II), and Cd(II) on nHAP-CGS can be described as a spontaneous, endothermic, and entropy-favored process governed jointly by entropy increase and chemical interactions between the adsorbent and the metal ions. In comparison with several representative adsorbents reported in the literature, nHAP-CGS exhibits higher adsorption capacities for Pb(II), Cu(II), and Cd(II), as summarized in Table 4, highlighting its potential as an efficient adsorbent for heavy metal removal.

**Table 4:** Comparison of properties of nFeS-CG and other adsorbents

Absorbent	*q*_*m*_ (mg/g)			pH	Dosage (g/L)	References
	Pb(II)	Cu(II)	Cd(II)			
nHAP-CGS	38.84	37.89	31.20	4	2.5	this study
Modified nano zeolite	–	17.92	18.32	7.5	2.0	Kamran Haghighi et al.^42^
Carbon nanotube adsorption materials	13.92	11.1	–	7	1.8	Abu Bakar et al.^43^
IT@SA	10.00	2.404	–	4	0.5	Xu et al.^44^
Utilization of activated biomass from ulva flexuosa	–	–	13.56	4	0.4	Lekshmi et al.^45^

### Effects of coexisting ions

**Figure 8:**
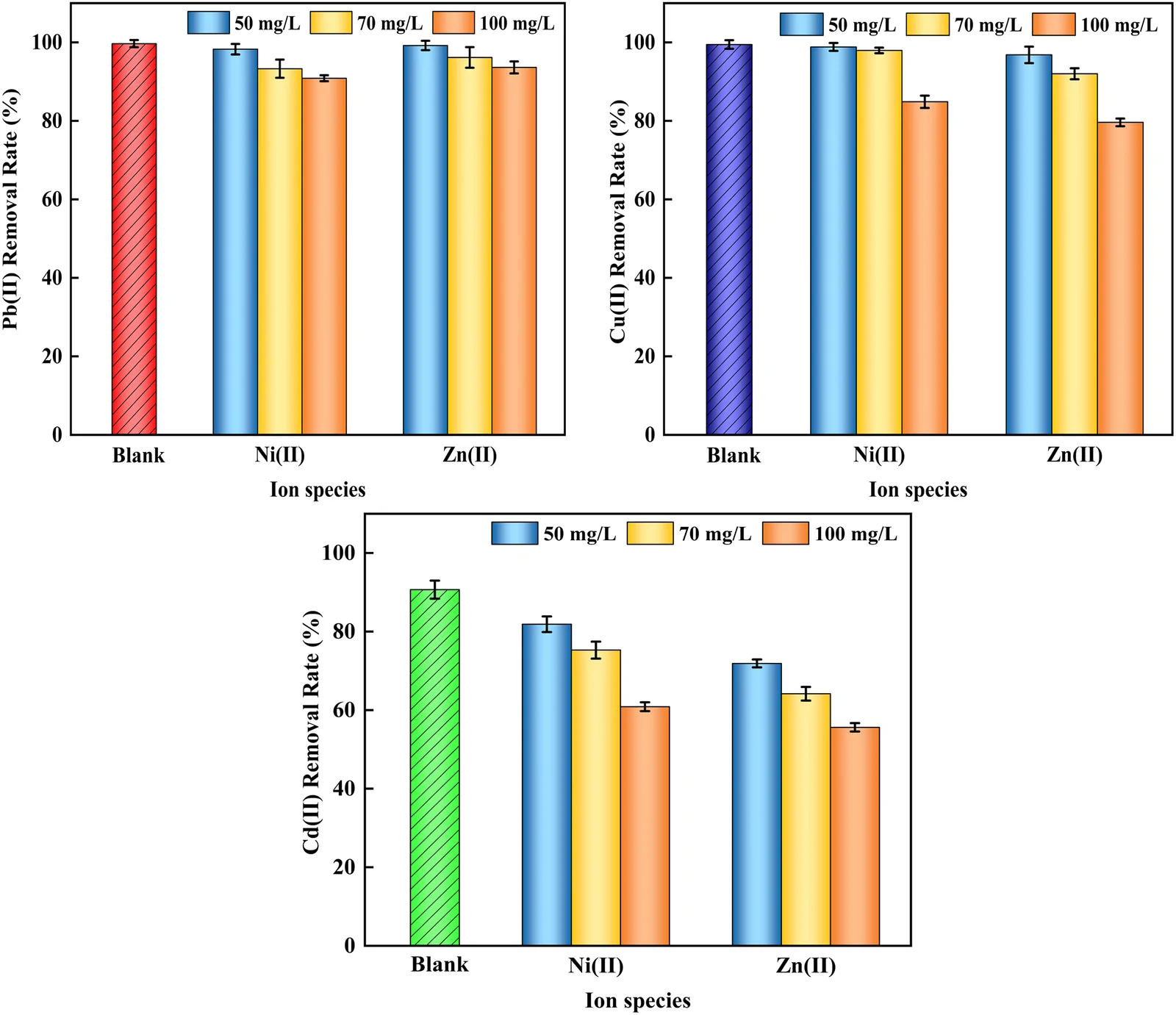
Removal rate of nHAP-CGS for Pb(II), Cu(II), and Cd(II) in the presence of Ni(II) and Zn(II) (all data are represented as mean ± SD)

The influence of coexisting ions on the simultaneous removal of Pb(II), Cu(II), and Cd(II) by nHAP-CGS was examined. In the absence of coexisting ions, an extremely high affinity of nHAP-CGS for Pb(II) is observed, with a removal rate of 99.68%. Once coexisting ions are introduced, the removal of Pb(II) is inhibited to varying extents. When the concentrations of Ni(II) or Zn(II) are varied from 50 to 100 mg/L, the Pb(II) removal rate decreases from 98.27% to 90.88% in the presence of Ni(II) and from 99.22% to 93.63% in the presence of Zn(II). Even under the highest interference level (100 mg/L), the removal of Pb(II) still exceeds 90%. This strong tolerance is mainly attributed to the formation of sparingly soluble lead phosphate precipitates on the nHAP surface and to the relatively high electronegativity of Pb(II), which favors the formation of stable inner-sphere complexes that are difficult to displace by competing ions.^46^ By contrast, the adsorption of Cu(II) is dominated by surface complexation and is more susceptible to competition from Zn(II) and Ni(II), which have similar chemical behavior; thus, significant inhibition is observed at elevated concentrations. Cd(II) exhibits the weakest resistance to coexisting ions. This can be ascribed to the higher solubility of its phosphate compounds, its lower electronegativity, and its particularly strong competition with the congeneric element Zn(II) for active sites.^25^ To further elucidate the competitive behavior, the removal efficiencies of the competing ions Zn(II) and Ni(II) themselves were measured at an initial concentration of 100 mg/L. The removal efficiencies of Zn(II) and Ni(II) were 76.34% and 70.25%, respectively, while Cd(II) removal was approximately 60.88% under the same conditions (Figure 8). Thus, the removal rate order under coexisting conditions was Pb(II) > Cu(II) > Zn(II) > Ni(II) > Cd(II). These results confirm that all tested ions compete for adsorption sites on nHAP-CGS, with Pb^2+^ showing the strongest affinity. The adsorption selectivity of nHAP-CGS for Pb(II), Cu(II), and Cd(II) follows the order Pb(II) > Cu(II) > Cd(II). This is consistently supported by the removal efficiencies (Figures 1, 2, 3, and 4), Langmuir maximum adsorption capacities (Table 1: 46.31 > 42.19 > 35.01 mg/g), and kinetic rate constants (Table 2). Even in the presence of competing ions (Zn(II) and Ni(II), Figure 8), Pb(II) removal remains above 90%, further confirming its preferential adsorption. This study only examined the competitive effects of Zn(II) and Ni(II). Other coexisting ions, such as Fe(III) and Mn(II), although common in lead-zinc mine wastewater, have not been studied in experiments on coexisting ions due to their mature removal techniques; Their potential impact on the performance of nHAP-CGS deserves further investigation.

### Leaching toxicity

The leaching toxicity of nHAP-CGS was assessed to evaluate the environmental safety of the material in water treatment applications. Leachates were analyzed by flame atomic absorption spectrophotometry in accordance with the Chinese national standards GB 5085.3–2007 and HJ/T299-2007. The concentrations of key heavy metals (Cr, Pb, Cu, Cd, Zn and Ni) in the leachates of CGS and nHAP-CGS are given in Table 5. For nHAP-CGS, the leaching concentrations of all heavy metals are far below the threshold values specified in the Identification Standards for Hazardous Wastes—Identification for Leaching Toxicity (GB 5085.3–2007) and are also markedly lower than those of the raw CGS. These results demonstrate that nano-hydroxyapatite modification endows the composite with a strong capacity to immobilize heavy metals.

**Table 5:** Heavy metal concentrations in coal Gangue and nHAP-CGS Leachate (mg/L)

Element	Mean		Leaching Toxicity standard value
	CGS	nHAP-CGS	
Cr	0.686	0.384	15
Pb	0.742	0.167	5
Cu	0.496	0.227	100
Cd	0.106	ND	1
Zn	0.283	0.149	100
Ni	0.079	ND	5

Under acidic leaching conditions, hydroxyapatite crystals supported on nHAP-CGS can be partially dissolved, releasing phosphate (PO_4_^3−^) and hydroxyl (OH^−^) groups into the solution. These reactive species readily react with dissolved metal ions to form poorly soluble metal phosphates and hydroxides, thereby stabilizing and immobilizing heavy metal ions within the solid matrix.^47^ This highly efficient fixation mechanism greatly reduces the mobility of heavy metals in aqueous environments and significantly improves the environmental safety of nHAP-CGS when used for water treatment. Consequently, nHAP-CGS can be regarded as a safe and effective adsorbent for the treatment of wastewater containing Pb(II), Cu(II), Cd(II), and other heavy metals.

### Cyclic regeneration

**Figure 9:**
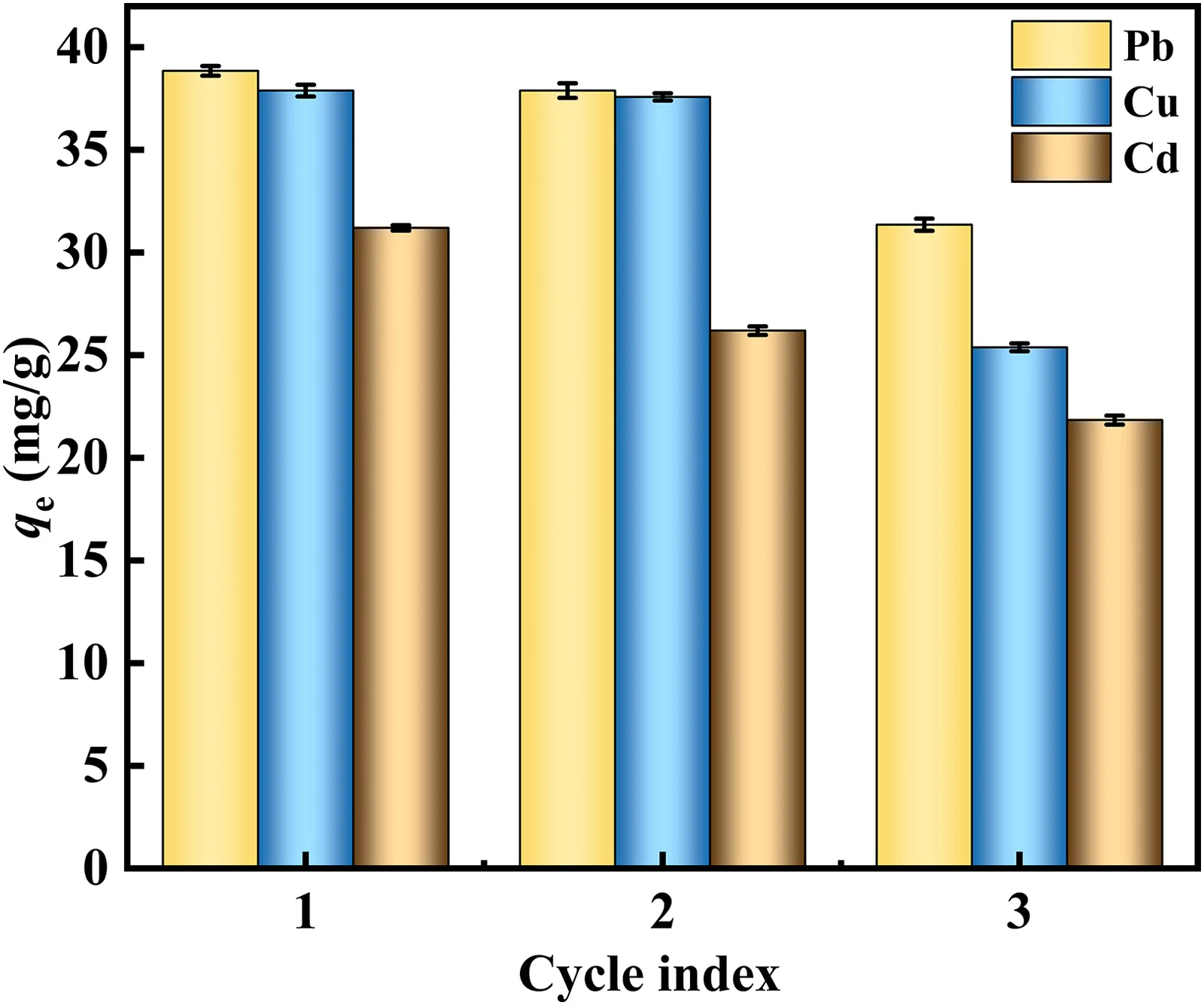
Cyclic regeneration experiment of nHAP-CGS adsorption of Pb(II), Cu(II), and Cd(II) (all data are represented as mean ± SD)

Cyclic adsorption-desorption experiments were conducted to investigate the regeneration and reusability of nHAP-CGS and to demonstrate the practicality and cost-effectiveness of the adsorbent in real wastewater treatment. As illustrated in Figure 9, the adsorption capacities of nHAP-CGS for Pb(II), Cu(II), and Cd(II) decrease progressively with increasing regeneration cycles, suggesting that repeated desorption-regeneration causes partial loss of active phases or structural changes, leading to a reduction in effective adsorption sites and a decrease in the content of the active hydroxyapatite phase.^48^ Even after three adsorption-desorption cycles, nHAP-CGS still maintains adsorption capacities of 31.36, 25.38 and 8.10 mg/g for Pb(II), Cu(II), and Cd(II), respectively. Based on these data, the regeneration efficiencies after three cycles were 80.7% for Pb(II), 67.0% for Cu(II), and 70.0% for Cd(II) (Table 6). These results indicate that nHAP-CGS possesses good regeneration performance and can be reused multiple times while retaining substantial adsorption capacity.

**Table 6:** Regeneration efficiencies of nHAP-CGS for Pb(II), Cu(II), and Cd(II) over three adsorption-desorption cycles

Number of cycles	Pb(II)	Cu(II)	Cd(II)
1	100%	100%	100%
2	97.5%	99.2%	84.05
3	80.7%	67.0%	70.0%

### Characterization analysis

#### Scanning electron microscopy (SEM)

**Figure 10:**
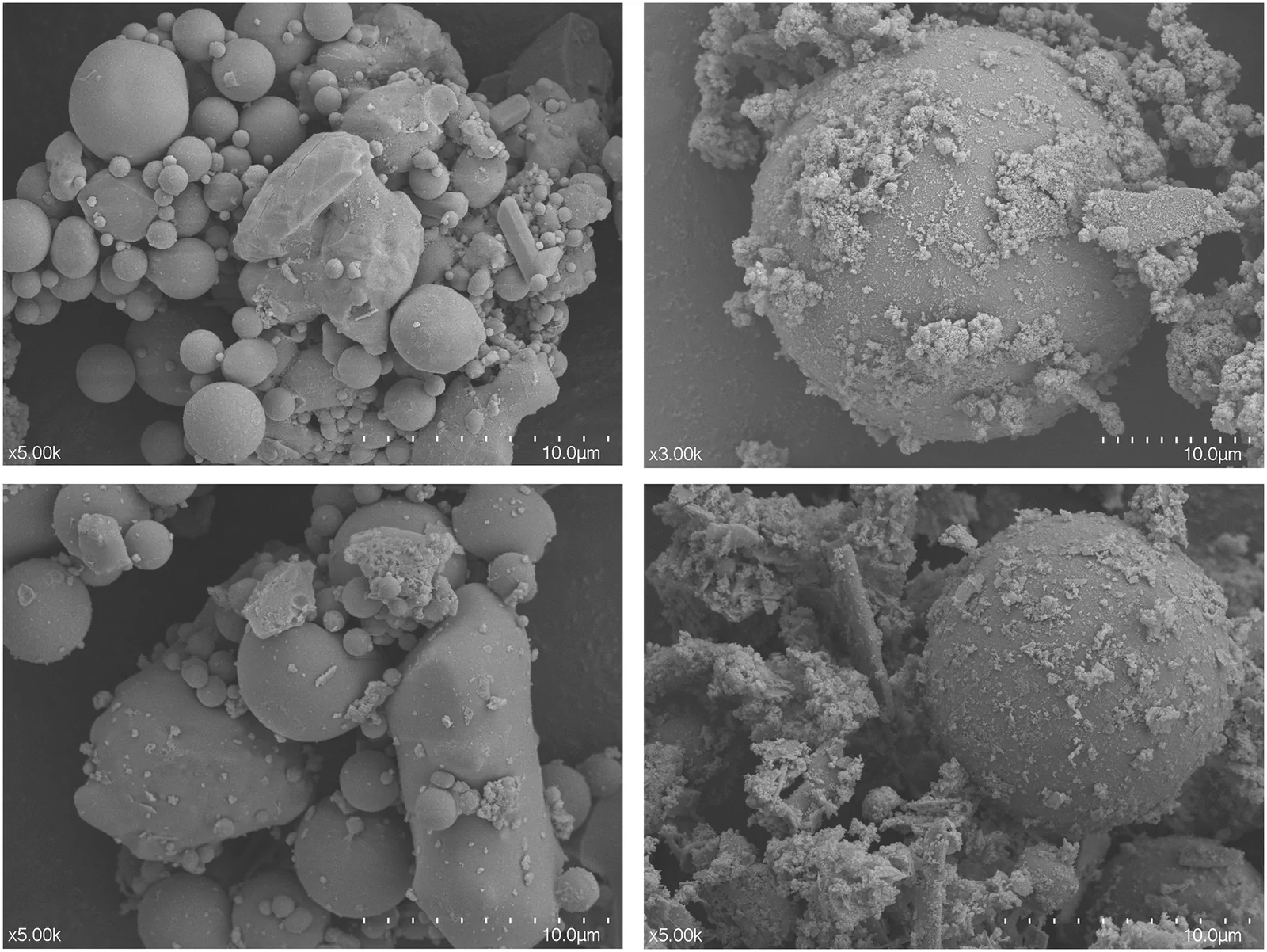
SEM images of coal gangue before and after adsorption with nHAP-CGS (scale bars represent 10.0 μm)

The morphological evolution of coal gasification slag and nHAP-CGS was examined by SEM (Figure 10). Before reaction, coal gasification slag is observed as irregular blocky or plate-like particles with relatively compact surfaces. Nevertheless, inter-particle regions and local surfaces contain discernible pores and fissures, forming a macroporous framework. After nHAP loading, the surface becomes markedly rougher and is uniformly decorated with numerous fine particles and protrusions, exhibiting a typical “nanoparticles anchored on a mineral skeleton” morphology. As a result, the specific surface area and density of potential active interfaces are greatly amplified, constructing a hierarchical structure favorable for heavy metal capture and immobilization.

Following exposure to acidic wastewater, the particle surfaces are covered by fine particulates or flocculent deposits that are preferentially enriched along pre-existing pores, grooves, and edges. This pattern is consistent with a pronounced dissolutionreprecipitation process of the matrix minerals under long-term acidic leaching.^49^ In the case of nHAP-CGS, the overall particle contours remain essentially intact compared with the uniformly distributed spherical nHAP particles before adsorption, indicating that nHAP maintains high structural stability in acidic conditions. The HAP sphere surfaces and interparticle gaps are partially obscured by dense accumulations of fine particles and flocculent deposits, and local “bridging” between adjacent particles by these deposits leads to a more compact and continuous deposition layer. This composite interfacial morphology, characterized by a “stable nano-skeleton plus a high-coverage deposited layer”, is highly consistent with a mechanism in which nHAP provides abundant phosphate and hydroxyl sites, thereby promoting heavy metal complexation, precipitation and co-precipitation.^50^ The high areal density of deposits on the nHAP-CGS surface suggests that Pb(II), Cu(II), and Cd(II) are immobilized on the surface, likely via precipitation or surface binding, rather than solely by physical adsorption. However, surface complexation occurs at the molecular/atomic level and cannot be directly observed by SEM. Direct evidence of surface complexation is provided by FTIR (formation of P–O–M bonds) in the Fourier transform infrared spectroscopy (FTIR) section.

#### X-ray diffraction (XRD)

**Figure 11:**
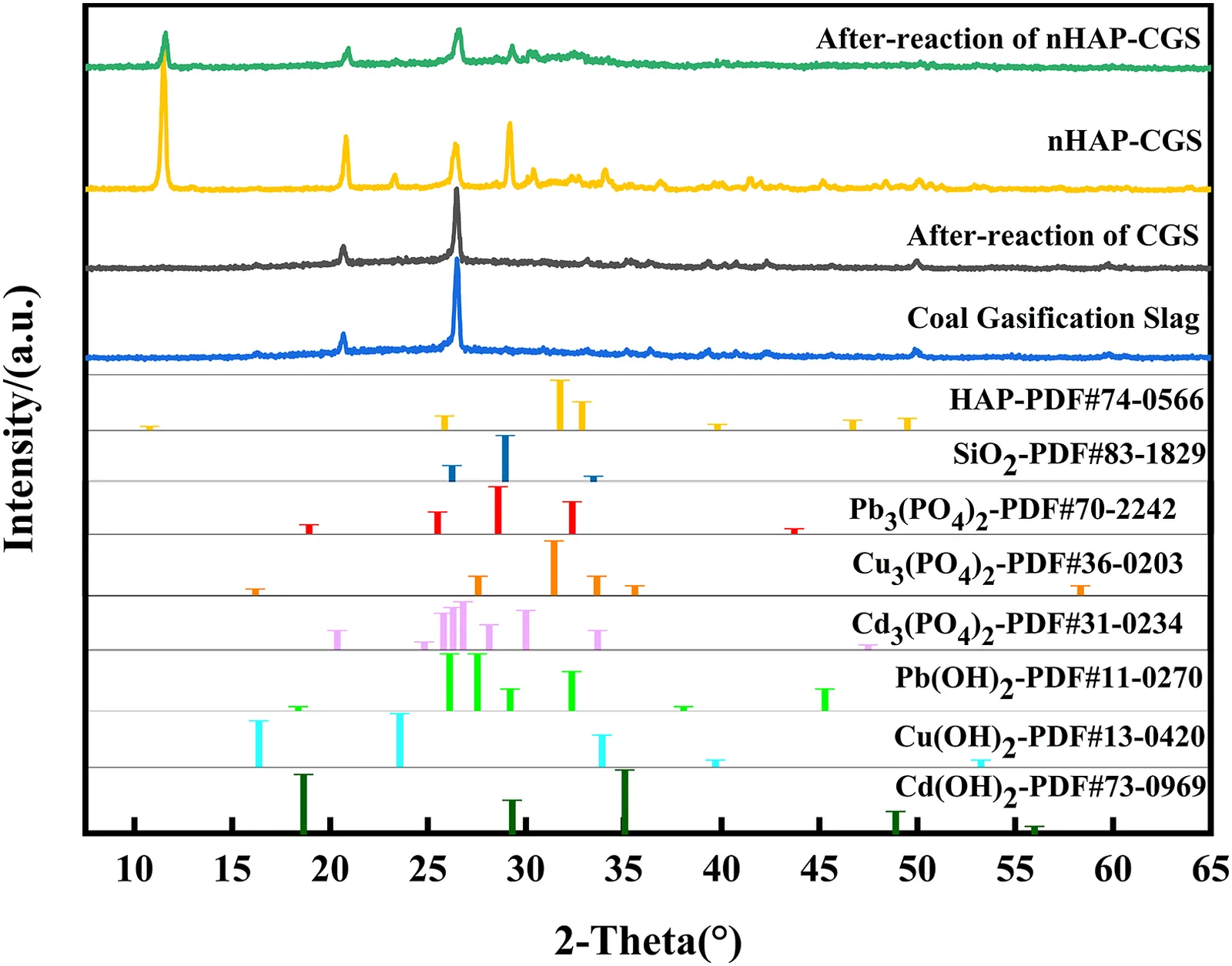
XRD diffraction patterns of coal gasification slag before and after reaction with nHAP-CGS

XRD patterns of coal gasification slag and nHAP-CGS before and after treatment of Pb(II), Cu(II), and Cd(II) bearing wastewater are presented in Figure 11.

For coal gasification slag, the XRD patterns before and after adsorption are nearly identical: the main peak remains the quartz reflection at 26.5°, and the positions of the other characteristic peaks change negligibly. Only slight variations in relative intensities are observed, and no new diffraction peaks are detected, indicating that no significant quantities of new crystalline phases are formed under the examined conditions. In contrast, nHAP-CGS exhibits a series of relatively sharp diffraction peaks at 11.5°, 20.8°, 23.3°, 26.4°, 29.2°, 30.1°–30.4°, 32.3°–32.7° and 34.0°. Peaks near 26.4°, 32.3°–32.7° and 34.0° can be indexed to the (002), (211)/(112) and (300) planes of hexagonal hydroxyapatite, confirming the successful loading of nHAP.^51^ After adsorption, the XRD pattern of nHAP-CGS shows pronounced intensity enhancement of peaks at 29.3°, 30.2°–30.5°, 31.1°–31.9°, 32.1°–32.9° and 33.2°, accompanied by peak broadening and slight shifts. The ionic radius of Pb^2+^ is larger than that of Ca^2+^, while Cu^2+^ and Cd^2+^ are smaller or comparable. The observed peak shifts and broadening are therefore consistent with the incorporation of these ions into the apatite lattice, which would cause local distortions and changes in unit-cell parameters depending on the substituting ion. Such lattice parameter changes are characteristic of cation exchange in apatite-type structures. These changes suggest that Pb^2+^, Cu^2+^, and Cd^2+^ may be incorporated into the apatite lattice, causing local distortions and unit-cell parameter variations, which is consistent with cation substitution (ion exchange). Low-angle reflections at 11.6° and 20.9° persist but with reduced relative intensity. These changes suggest the occurrence of dissolutionreprecipitation or secondary crystallization during heavy metal uptake. Partial substitution of Ca(II) by Pb(II), Cu(II), and Cd(II) in the apatite lattice is likely, leading to subtle adjustments of the unit-cell parameters and, consequently, to modifications in peak positions and relative intensities within the 29°–34° range.^52^ Under PO_4_^3−^ rich conditions, Pb(II) is further prone to form thermodynamically stable lead-apatite-type phases whose characteristic peaks overlap strongly with those of hydroxyapatite in the 2θ range of 29°–33°. As a result, this region displays markedly enhanced, split, and broadened peaks.^53^ For Cu(II) and Cd(II), the formation of poorly crystalline metal phosphates or Ca–P solid solutions is also plausible, and these phases similarly manifest as slight shifts of the main apatite reflections and the appearance of shoulder peaks.

#### Fourier transform infrared spectroscopy (FTIR)

**Figure 12:**
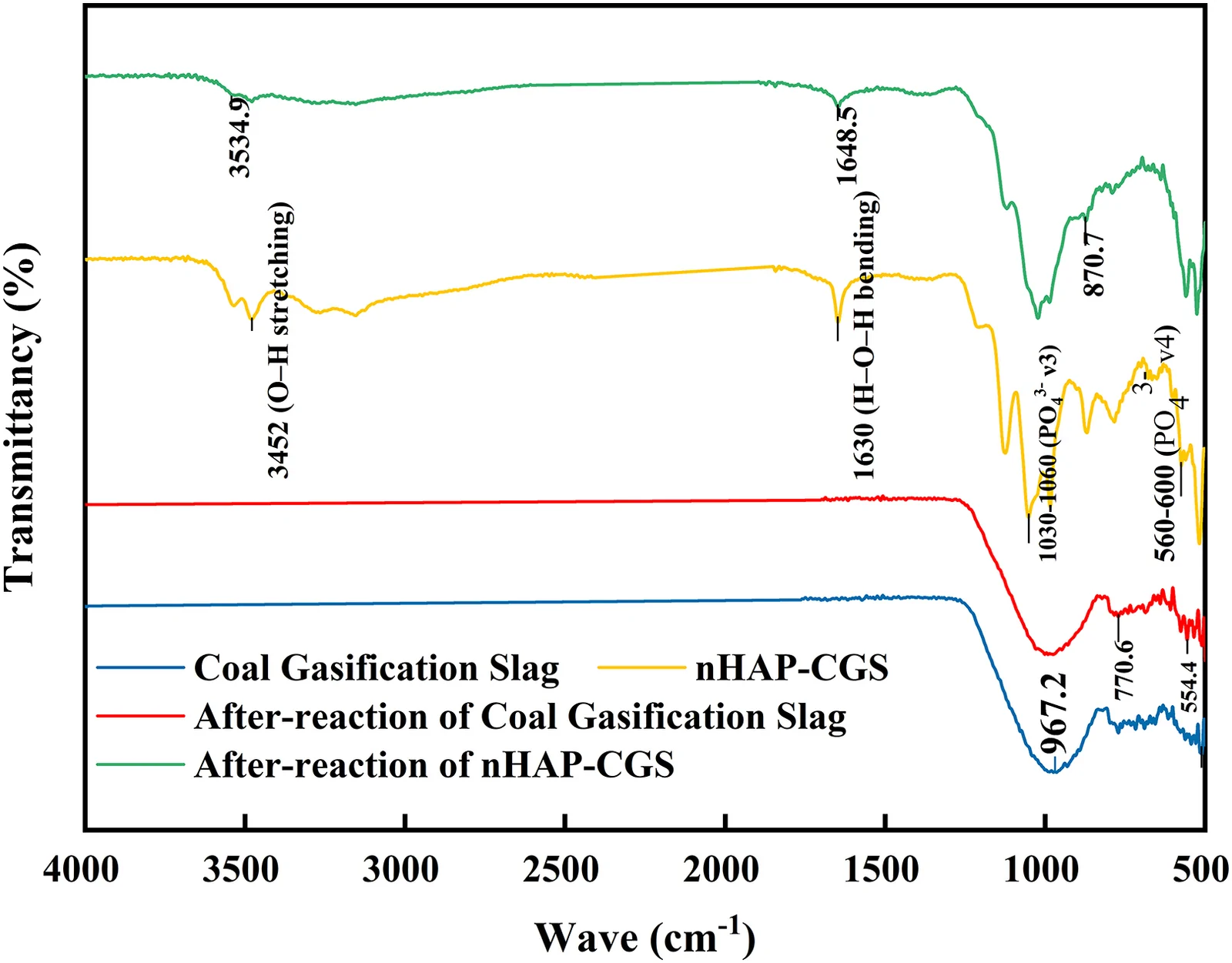
FTIR spectra of coal gasification slag before and after reaction with nHAP-CGS

The FTIR spectra of coal gasification slag and nHAP-CGS before and after adsorption are shown in Figure 12. For coal gasification slag, the spectra before and after heavy metal uptake are largely similar, with differences mainly in band intensities. The –OH band at 3452 cm^−1^ becomes weaker, indicating partial degradation of structural units such as Si–O and Al–OH under acidic conditions. The Si–O–Si/Al stretching band in the 1000–1100 cm^−1^ region is also attenuated, suggesting partial decomposition of quartz.^54^ These observations indicate that the adsorption of Pb(II), Cu(II), and Cd(II) on raw CGS is primarily governed by physical interactions (e.g., electrostatic attraction, pore diffusion), with only minor contributions from chemical interactions due to the limited number of active sites on the inert aluminosilicate surface.

For nHAP-CGS, more pronounced spectral evolution is observed. After adsorption, the PO_4_^3−^ ν_3_ stretching band in the 1030–1060 cm^−1^ region exhibits a red shift and broadening, with the band maximum moving to lower wavenumbers. This behavior indicates that the local electronic environment of PO_4_ tetrahedra is modified by the formation of P–O–M (M = Pb, Cu, Cd) bonds, consistent with partial Ca^2+^ substitution by heavy metal ions and dissolution-reprecipitation of Ca–P phases.^55^ The red shift of the PO_4_^3−^ stretching band indicates a weakening of the P–O bond strength, which is expected when Ca^2+^ is replaced by metal ions with different electronegativity and coordination preferences. The formation of P–O–M bonds (M = Pb, Cu, Cd) alters the local electronic environment of the phosphate tetrahedra, consistent with the incorporation of metal ions into the apatite structure via ion exchange. The ν_4_ bending doublet of PO_4_^3−^ at 560–600 cm^−1^ also changes in intensity ratio and shape, and some bands extend into the 510–530 cm^−1^ region, indicating M–O–P or M–O bending vibrations in newly formed metal phosphate structures and implying the generation of Pb–P, Cu–P and Cd–P linkages.^56^ These spectral changes (red shift of the PO_4_^3−^ ν_3_ band and alterations in the ν_4_ bending doublet) are consistent with the formation of P–O–M bonds, which may arise from Ca^2+^ being replaced by heavy metal ions via ion exchange. The high-wavenumber O–H stretching band (3200–3600 cm^−1^) and the H–O–H bending band (1630 cm^−1^) both show significant changes in intensity and width, suggesting that lattice hydroxyls and adsorbed water are actively involved in precipitation and surface complexation. These species are partially consumed and reorganized within a newly formed hydrogen-bond network during dissolution-recrystallization.^57^ In the 1400–1500 cm^−1^ region, enhancement of weak bands and more distinct shoulder features are observed, which can be associated with increased CO_3_^2−^ incorporation or the generation of carbonated heavy-metal apatites. Compared with coal gasification slag, the FTIR evolution of nHAP-CGS is more systematic, with both O–H and PO_4_^3−^ bands undergoing coherent shifts and shape changes. This contrast reflects a fundamental difference in the heavy metal removal mechanisms of the two materials.

#### BET surface area and pore structure

**Figure 13:**
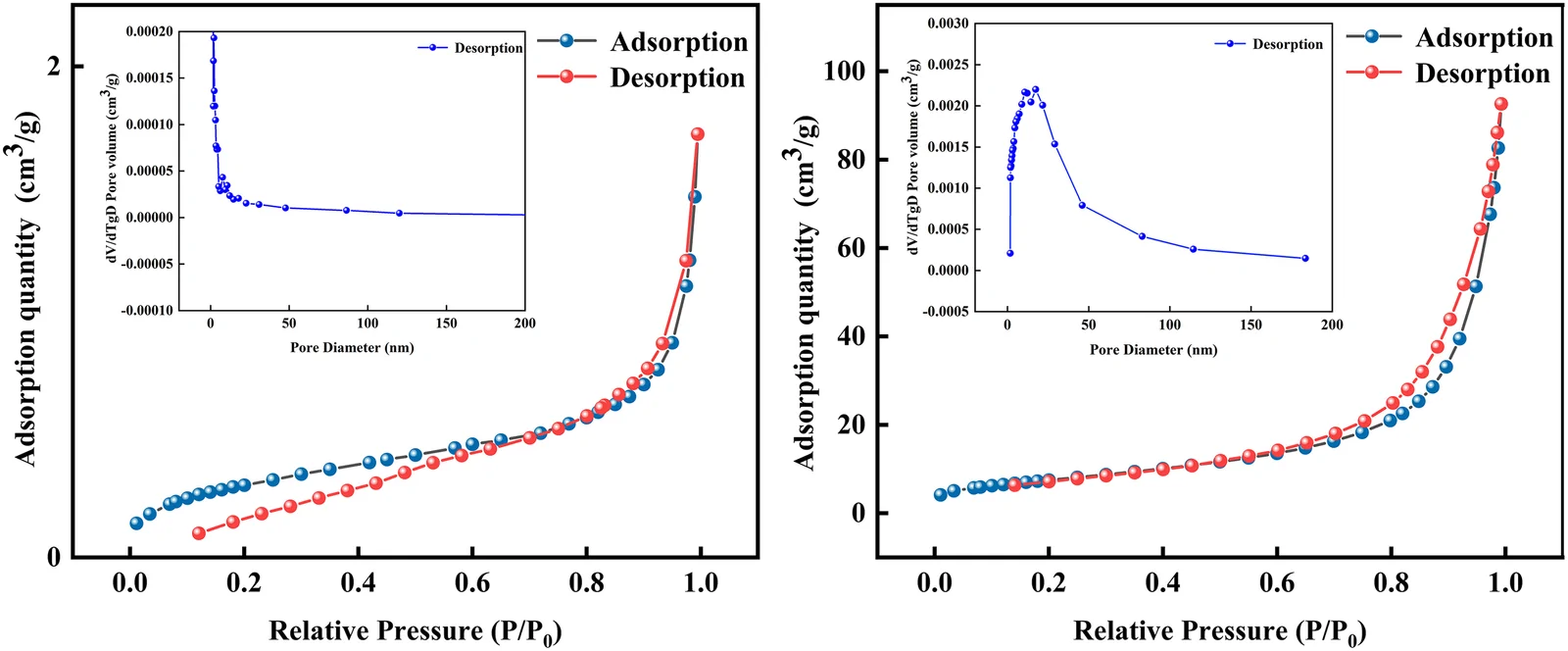
N_2_ adsorption-desorption isotherm curves and pore size distribution curves of coal gasification slag and nHAP-CGS

N_2_ adsorption-desorption isotherms and pore-size distributions for coal gasification slag and nHAP-CGS are presented in Figure 13, and the corresponding textural parameters are summarized in Table 7. Coal gasification slag exhibits a type II isotherm with an H_3_ hysteresis loop, indicating the presence of slit-like macropores and mesopores. In contrast, nHAP-CGS displays a type IV isotherm with an H_3_ hysteresis loop that does not close completely, suggesting slit-shaped or irregular pores associated with plate-like or layered structures. These features confirm that nHAP-CGS is a mesoporous material with a relatively high surface area and pore volume, likely formed by the stacking of plate-like particles that generate slit-shaped voids.

**Table 7:** Parameters of specific surface area, pore volume, and pore size for coal gangue and nHAP-CGS

Name	Specific surface area (m^2^/g)	Confucius (cm^3^/g)	Aperture (nm)
CGS	3.092	0.0025	19.46
nHAP-CGS	57.65	0.245	13.3

The specific surface areas of coal gasification slag and nHAP-CGS are 3.092 and 57.65 m^2^/g, respectively, representing a 52.79-fold increase upon nHAP modification. The pore volumes increase from 0.025 to 0.245 cm^3^/g, indicating a much more developed pore network in nHAP-CGS. The dominant pore size decreases from 125.51 nm for coal gasification slag to 10.52 nm for nHAP-CGS, implying that nano-HAP particles are deposited on the slag surface and within its pores, partially filling pre-existing mesopores and macropores and thereby reducing the average pore size.^58^ The pore-size distribution of nHAP-CGS is mainly concentrated between 3 and 10 nm, further confirming its mesoporous character and the creation of abundant accessible sites for heavy metal adsorption.

### Adsorption mechanism

The adsorption mechanism of nHAP-CGS for Pb(II), Cu(II), and Cd(II) in lead-zinc mine wastewater was clarified by integrating performance evaluation, adsorption kinetics, isotherms, thermodynamics, and multi-scale characterization. Both surface complexation and precipitation contribute to the chemical adsorption. For Pb(II), precipitation of lead phosphate is dominant; for Cu(II) and Cd(II), surface complexation plays a relatively more important role due to the higher solubility of their phosphates. BET analysis revealed that, after nHAP loading, the specific surface area and pore volume of CGS were substantially increased to the order of 10^2^ m^2^ g^−1^, with pore sizes concentrated in the 3–10 nm mesoporous range. This textural evolution provides abundant reactive interfaces and efficient mass-transfer pathways for ion exchange, surface complexation, and precipitation reactions. SEM observations confirmed that the nHAP-CGS surface was uniformly decorated with nano-sized particles, and that dense, fine deposits formed on the surface after adsorption. In the XRD patterns, enhancement, broadening and slight shifts of the characteristic nHAP-CGS peaks indicated partial substitution of Ca^2+^ by heavy-metal ions, giving rise to Ca–M coherent solid-solution structures.^59^ In FTIR spectra, shifts and broadening of the PO_4_^3−^ vibration bands (ν_3_ and ν_4_ regions) further demonstrated perturbation of the local electronic environment of the crystal lattice by heavy-metal substitution, confirming that phosphate groups (PO_4_^3−^) are directly involved in metal coordination via P–O–M bond formation. Meanwhile, variations in the O–H stretching band (3200–3600 cm^−1^) indicate that surface hydroxyl groups (OH^−^) also participate in surface complexation, suggesting that Ca^2+^–M^2+^ ion exchange may contribute to the overall adsorption process. The gradual increase in solution pH during adsorption, together with the variations in O–H stretching and H–O–H bending bands in the FTIR spectra, suggests that lattice hydroxyls and surface-adsorbed water molecules participated in metal coordination and precipitation. Deprotonated surface groups such as ≡PO_4_^−^ and ≡M–OH are able to form inner-sphere complexes with M^2+^; this inner-sphere complexation is consistent with the pseudo-second-order kinetic behavior and with the adsorption-energy evolution described by the Temkin model. Because Cd phosphates exhibit higher solubility and lower precipitation stability, Cd(II) removal is more strongly dependent on a combination of physical adsorption, outer-sphere complexation, and limited ion exchange. Consequently, both the adsorption capacity and the resistance to coexisting-ion interference for Cd(II) are lower than those for Pb(II) and Cu(II).

On the basis of adsorption isotherm, kinetic and thermodynamic analyses, and supported by BET, XRD, FTIR and SEM results, it is concluded that Pb(II), Cu(II), and Cd(II) removal by nHAP-CGS is dominated by chemisorption, assisted by physical adsorption and pore-channel entrapment, and that a pronounced selectivity of Pb(II) > Cu(II) > Cd(II) is exhibited. The porous CGS skeleton serves as a mass-transfer network, whereas the loaded nHAP provides a high density of PO_4_^3−^/OH^−^ functional groups and nano-scale reaction interfaces. Through this structural complementarity, a coupled multi-mechanistic adsorption platform integrating ion exchange, surface complexation, precipitation/co-precipitation, and pore-channel entrapment is established.

### Conclusions

A coal gasification slag-supported nano-hydroxyapatite composite (nHAP-CGS) was successfully prepared by combining a loading strategy with chemical precipitation, exploiting the mechanically stable mineral framework of coal gasification slag and the strong affinity of nano-hydroxyapatite for heavy-metal ions. The composite was employed to remove Pb(II), Cu(II), and Cd(II) from lead–zinc mine wastewater. In the performance evaluation experiments, the effects of adsorbent dosage, initial pH, contact time, and initial metal concentration on removal performance were systematically assessed. Under optimized conditions (dosage 2.5 g L^−1^, initial pH 4, contact time 120 min and initial concentration 100 mg L^−1^), removal efficiencies of 97.6%, 94.9% and 79.8% were achieved for Pb(II), Cu(II), and Cd(II), respectively, significantly outperforming the unmodified slag and revealing a clear selectivity sequence of Pb>Cu>Cd.

Adsorption isotherm and kinetic analyses showed that the uptake of these metal ions by nHAP-CGS followed the Langmuir monolayer isotherm and the pseudo-second-order kinetic model, with correlation coefficients (R^2^) generally exceeding 0.97. These results indicate that chemisorption is the predominant mechanism, with intra-particle diffusion contributing to the rate control. When combined with BET, XRD, FTIR, and SEM-EDS characterizations, the adsorption mechanism is attributed to the synergistic action of the enlarged specific surface area of the porous CGS framework and the PO_4_^3−^/OH^−^ active sites on the nHAP-CGS surface, which facilitate Ca^2+^–heavy-metal ion exchange, surface complexation, and metal phosphate/hydroxide precipitation-co-precipitation.

Coexisting-ion and leaching-toxicity tests demonstrated that nHAP-CGS maintains high removal efficiencies in complex multi-ion environments and that the leached heavy-metal concentrations remain far below the regulatory thresholds for hazardous waste, indicating good environmental safety. Moreover, appreciable adsorption capacity is retained after several adsorption-desorption cycles, confirming the regeneration and reusability of the material. Overall, nHAP-CGS prepared using coal gasification slag as a carrier represents a simple, high-performance, and environmentally benign adsorbent, offering a viable route and technical support for the simultaneous removal of multiple metal ions from lead-zinc mine wastewater and for the high-value utilization of coal-derived solid wastes.

### Limitations of the study

Several limitations of this study should be acknowledged. First, the coexisting-ion experiments focused primarily on Zn(II) and Ni(II), while other common ions in lead-zinc mine wastewater, such as Fe(III) and Mn(II), were not examined. Second, zeta potential measurements were not performed; instead, literature data were used to support the pH-dependent electrostatic interaction discussion. Third, the sampling points in the kinetic experiments started from 10 min; denser sampling within the first 10 min would refine the intra-particle diffusion model fitting. Finally, competitive adsorption was analyzed based on removal efficiencies rather than full binary or ternary isotherm models (e.g., Extended Langmuir), which could be pursued in future studies. Despite these limitations, the experimental data sufficiently support the main conclusions regarding the synergistic adsorption performance of nHAP-CGS.

## Resource availability

### Lead contact

Further information and requests for resources and reagents should be directed to and will be fulfilled by the Lead Contact, Xuying Guo (guoxuying@lntu.edu.cn).

### Materials availability

This study did not generate new unique reagents.

### Data and code availability

• Data reported in this paper will be shared by the lead contact upon request.
• This study did not generate any code.
• Any additional information required to reanalyze the data reported in this paper is available from the lead contact upon request.

## Acknowledgments

The work is funded by the National Natural Science Foundation of China (51304114, 52304188), the Department of Education of Liaoning Province (LJKFZ20220199), Science and Technology Department of Liaoning Province (2023-BS-201).

## Author contributions

X.G.: conceptualization, methodology, validation, formal analysis, and writing – original draft preparation. F.M.: software, resources, data curation, visualization, and writing—review and editing. X.Z.: conceptualization, investigation, and writing – original draft preparation. W.S.: investigation, project administration, and supervision. Y.D.: conceptualization, methodology, resources, and supervision.

## Declaration of interests

The authors declare no competing interests.

## STAR★Methods

### Key resources table

**Table:** 

REAGENT or RESOURCE	SOURCE	IDENTIFIER
**Chemicals, peptides, and recombinant proteins**		

Pb(NO_3_)_2_	Sinopharm Chemical Reagent	CAS 10099-74-8
Cu(NO_3_)_2_·3H_2_O	Sinopharm Chemical Reagent	CAS 10031-43-3
Cd(NO_3_)_2_·4H_2_O	Sinopharm Chemical Reagent	CAS 10022-68-1
Ca(NO_3_)_2_	Sinopharm Chemical Reagent	CAS 10124-37-5
Na_2_HPO_4_·12H_2_O	Sinopharm Chemical Reagent	CAS 10039-32-4
EDTA	Sinopharm Chemical Reagent	CAS 60-00-4

**Software and algorithms**		

Origin 2021	OriginLab	RRID: SCR_014212
MDI Jade 6.0	MDI	RRID: SCR_012800

**Other**		

Flame Atomic Absorption Spectrometer	GB 7475-87	N/A
XRD (Bruker D8 Advance)	Bruker	N/A
FTIR (ALPHA II)	Bruker	N/A
BET (ASAP 2460)	Micromeritics	N/A

### Method details

#### Materials

Coal gasification slag (CGS) used in this study was collected from Chifeng, Inner Mongolia, China (42.01°N, 121.65°E). The CGS was sieved to obtain a particle size fraction of 0.045–0.075 mm, washed three times with deionized water, and dried in a blast oven (DHG-9030, Shanghai Yiheng Scientific Instrument Co., Ltd., China) at 378.15 K before use. The main chemical composition of the CGS was determined by X-ray fluorescence spectroscopy (XRF). The following reagents were used: NaOH, HNO_3_, Ca(NO_3_)_2_, Na_2_HPO_4_·12H_2_O, Pb(NO_3_)_2_, Cu(NO_3_)_2_·3H_2_O, and Cd(NO_3_)_2_·4H_2_O. All reagents were of analytical grade and were purchased from Sinopharm Chemical Reagent Co., Ltd. (Shanghai, China). Deionized water was used throughout all experiments.

Main compositions of Fuxin coal gangue.

**Table:** 

Ingredients	SiO_2_	TiO_2_	Al_2_O_3_	Fe_2_O_3_	MnO_2_	MgO	CaO	Na_2_O	K_2_O	P_2_O_5_	SO_3_
Content (%)	52.54	1.25	18.33	9.90	0.29	3.40	8.431	1.48	1.42	0.44	0.32

Simulated wastewater was prepared by setting heavy metal ion mass concentrations according to the actual water quality of a coal mine area. Pb(NO_3_)_2_, Cu(NO_3_)_2_·3H_2_O and Cd(NO_3_)_2_·4H_2_O were dissolved in deionized water to yield a solution containing Pb(II), Cu(II) and Cd(II) at 100 mg/L each. The pH was adjusted to 4 with 0.1 mol/L HNO_3_ and 0.1 mol/L NaOH.

#### Preparation of nHAP-CGS

Pretreated coal gasification slag (CGS, 5.0 g, 0.045–0.075 mm) was dispersed in 100 mL of 0.6 mol/L Ca(NO_3_)_2_ solution and stirred at 150 rpm using a magnetic stirrer (84-1, Suzhou Guohua Instrument Co., Ltd., China) for 40 min. Then, 100 mL of 1 mol/L Na_2_HPO_4_·12H_2_O solution was added. While stirring, 1 mol/L NaOH solution was added dropwise until the suspension reached pH 10.5.^60^ After a further 2 h of stirring, the mixture was aged at 25°C for 24 h in a constant-temperature water bath (SHA-8, Jiangsu Guowang Instrument Co., Ltd., China). The solid was recovered by filtration, washed to neutral pH, dried at 80°C in an electric blast oven (DHG-9030A, Shanghai Yiheng Scientific Instrument Co., Ltd., China), and ground to obtain the nHAP-CGS adsorbent (below figure).

**Figure:**
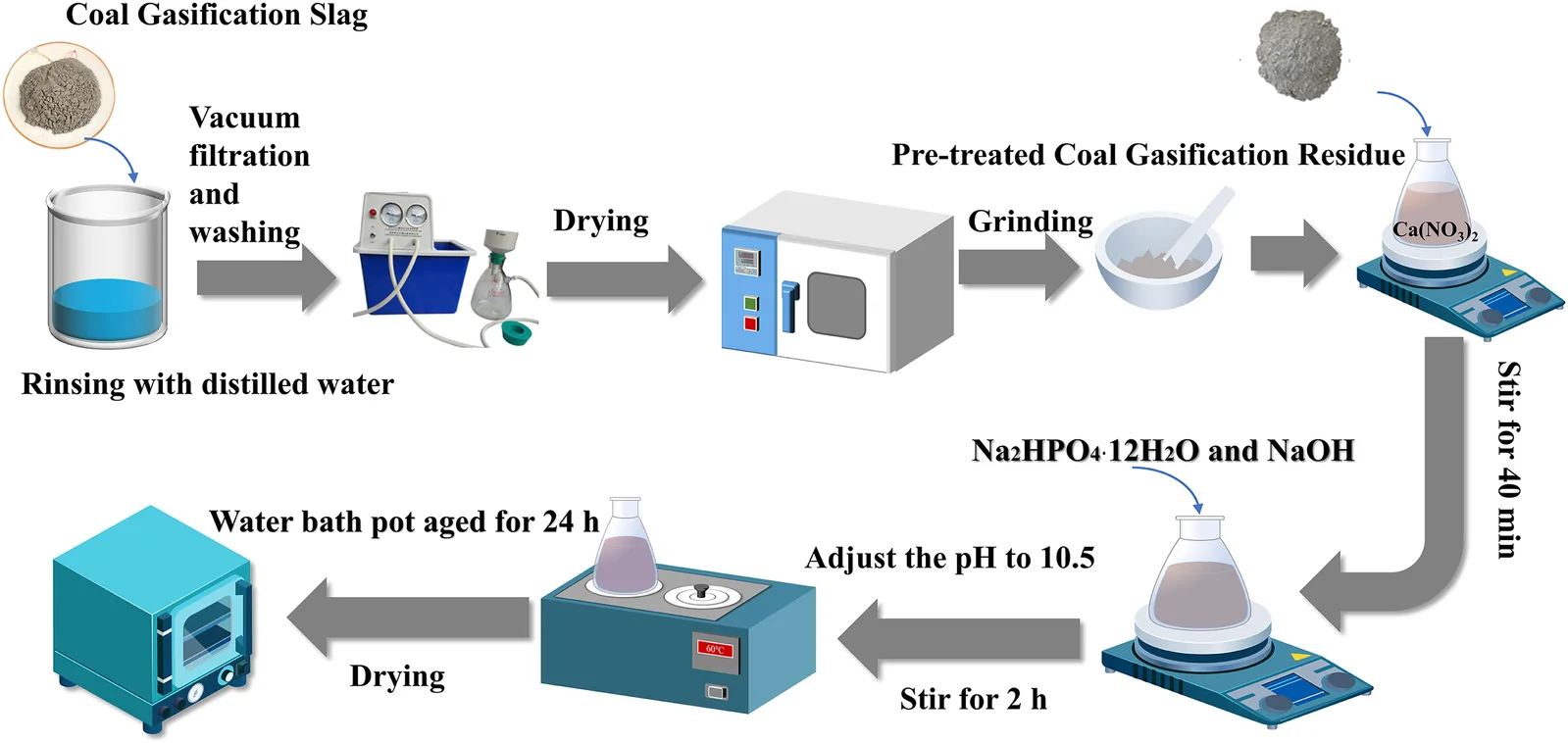
Preparation process of nHAP-CGS

#### Batch adsorption experiments

The adsorption behavior of nHAP-CGS toward Pb(II), Cu(II), and Cd(II) was evaluated in batch mode. The effects of adsorbent dosage, initial pH, contact time, and initial concentration were examined. Raw CGS (0.045–0.075 mm) at the same mass was used as a control. All experiments were performed in triplicate and mean values were used. removal rate η (%) and adsorption capacity q_e_ (mg/g) were calculated by Equations 1 and 2.

(Equation 1) $$\begin{array}{c} \eta =\frac{C_{0}-C_{t}}{C_{0}}\times 100\% \end{array}$$

(Equation 2) $$\begin{array}{c} q_{e}=\frac{\left(C_{0}-C_{t}\right)\times V}{m} \end{array}$$

In these equations, *η* denotes the removal rate of Pb(II), Cu(II) and Cd(II) (%); *C*_*0*_ is the initial concentration of the target ion (mg/L); *C*_*t*_ is the residual concentration of the ion after adsorption (mg/L); *q*_*e*_ is the adsorption capacity (mg/g); V is the solution volume (L); and m is the mass of the adsorbent (g).

To study the dosage effect, 2–6 g/L of nHAP-CGS was added to 200 mL of simulated wastewater (Pb(II), Cu(II), Cd(II) each 100 mg/L, pH 4, 298.15 K) and stirred at 160 rpm for 120 min. The influence of pH was investigated at pH 2–6 with 2.5 g/L nHAP-CGS (200 mL, 100 mg/L of each metal, 298.15 K, 160 rpm, 120 min). Contact time effects were assessed at 5–180 min under fixed conditions (2.5 g/L nHAP-CGS, 200 mL, 100 mg/L of each metal, pH 4, 298.15 K, 160 rpm). The influence of initial concentration was examined at 50–250 mg/L of each metal (2.5 g/L nHAP-CGS, 200 mL, pH 4, 298.15 K, 160 rpm, 120 min).

#### Isotherms, kinetics and thermodynamics

##### Isotherms

Isotherm experiments were conducted at 298.15 K and pH 4 with initial metal concentrations of 50–250 mg/L (200 mL, 2.5 g/L nHAP-CGS, 160 rpm, 120 min). After filtration (0.45 μm), residual metal concentrations were determined. Triplicate tests were averaged. Langmuir, Freundlich and Temkin models [Equations 3, 4, and 5]^61^ were used to fit adsorption of Pb(II), Cu(II), and Cd(II) on CGS and nHAP-CGS.

(Equation 3) $$q_{e}=\frac{q_{m}K_{L}C_{e}}{1+K_{L}C_{e}}$$

(Equation 4) $$q_{e}=K_{F}C_{e}^{1/n}$$

(Equation 5) $$\ln q_{e}=\ln K_{F}+\frac{1}{n}\ln C_{e}$$

In these equations, *C*_*e*_ denotes the equilibrium concentration of the pollutant in solution (mg/L); *q*_*e*_ denotes the equilibrium adsorption capacity of the adsorbent for the pollutant (mg/g); *q*_*m*_ denotes the saturated adsorption capacity of the adsorbent for the pollutant (mg/g); *K*_*L*_ denotes the Langmuir adsorption constant; *K*_*F*_ denotes the Freundlich adsorption constant; and n denotes the constant related to adsorption intensity.

##### Kinetics

Kinetic experiments were carried out at 298.15 K, pH 4, and 100 mg/L of each metal (200 mL, 2.5 g/L nHAP-CGS, 160 rpm). Samples were taken from 10 to 180 min and analyzed for residual Pb(II), Cu(II), and Cd(II). Triplicate runs were averaged. Pseudo-first-order, pseudo-second-order and intra-particle diffusion models [Equations 6, 7, and 8]^62^ were employed for kinetic fitting.

(Equation 6) $$\frac{t}{q_{t}}=\frac{1}{k_{2}q_{e}^{2}}+\frac{t}{q_{e}}$$

(Equation 7) $$q_{t}=k_{p}t^{1/2}+C$$

(Equation 8) $$q_{e}=\frac{RT}{b}\ln\left(aC_{e}\right)$$

In these equations, *q*_*t*_ denotes the adsorption capacity of the adsorbent for the pollutant at time ttt (mg/g); *q*_*e*_ denotes the adsorption capacity of the adsorbent for the pollutant at equilibrium (mg/g); *k*_*1*_ denotes the pseudo-first-order adsorption rate constant (min^−1^); *k*_*2*_ denotes the pseudo-second-order adsorption rate constant (mg/(g·min)); *k*_*p*_ denotes the intra-particle diffusion rate constant (mg/(g·min^1^ᐟ^2^)); *T* denotes the absolute temperature at which adsorption occurs (K); R denotes the ideal gas constant with a value of 8.314 J/(mol·K); and *C*_*e*_ denotes the equilibrium pollutant concentration (mg/L).

##### Thermodynamics

Thermodynamic tests were performed at 298.15, 308.15 and 318.15 K (pH 4, 100 mg/L of each metal, 200 mL, 2.5 g/L nHAP-CGS, 160 rpm, 120 min). Equilibrium concentrations were used to calculate K_d and thermodynamic parameters ΔG, ΔH and ΔS according to Equations 9, 10, 11, and 12,^63^

(Equation 9) $$K_{d}=\frac{q_{e}}{c_{e}}$$

(Equation 10) ΔG=-RTlnK

(Equation 11) ΔG=ΔH-TΔS

(Equation 12) $$\ln\;K_{d}=\frac{\Delta S}{R}\;-\;\frac{\Delta H}{RT}$$

In these equations, *ΔG* denotes the change in Gibbs free energy (kJ/mol); *ΔH* denotes the change in enthalpy (kJ/mol); *ΔS* denotes the change in entropy (J/(mol·K)); *K*_*d*_ denotes the thermodynamic equilibrium constant; *R* denotes the ideal gas constant (8.314 J/(mol·K)); and *T* denotes the temperature in Kelvin (K).

#### Coexisting-ion experiments

Coexisting-ion effects on the simultaneous removal of Pb(II), Cu(II), and Cd(II) by nHAP-CGS were evaluated. Based on field data, Zn(II) and Ni(II) were selected as representative coexisting ions due to their relatively high concentrations and hydrated radii similar to those of Cu(II) and Cd(II). Concentrations of 50, 70 and 100 mg/L were used. Simulated wastewater containing 100 mg/L of Pb(II), Cu(II), and Cd(II) was mixed with each coexisting ion separately, and a blank without coexisting ions was prepared. nHAP-CGS (2.5 g/L) was added, and suspensions were stirred at 160 rpm and 25°C for 120 min. After filtration (0.45 μm), residual Pb(II), Cu(II), and Cd(II) concentrations were measured. All tests were run in triplicate. The residual concentrations of Zn(II) and Ni(II) were determined by flame atomic absorption spectrometry (AAS), following the same procedure as described in the Analysis and characterization section..

#### Leaching toxicity

Leaching toxicity of CGS and nHAP-CGS was assessed according to HJ/T 300–2007. An acid leachant (pH 3.1–3.3) was prepared from H_2_SO_4_ and HNO_3_ (2:1, mass) and deionized water. Dried CGS and nHAP-CGS were added at a solid–liquid ratio of 1:10 g/mL and agitated for 18 h at room temperature. Heavy metals in the leachates (Cr, Pb, Cu, Cd, Zn, Ni) were determined by flame AAS following GB 5085.3–2007 and HJ/T 299–2007.

#### Regeneration

Regeneration performance of nHAP-CGS was evaluated by three successive adsorption–desorption cycles. For each cycle, 2.5 g/L of nHAP-CGS was contacted with 200 mL of simulated wastewater (Pb(II), Cu(II), Cd(II) each 100 mg/L) at 230 rpm and room temperature for 120 min. The saturated adsorbent was then desorbed with 0.5 mol/L EDTA for 3 h, centrifuged, washed, dried and reused under identical conditions.EDTA was selected as the desorption agent because of its strong chelating ability with Pb(II), Cu(II), Cd(II), forming stable water-soluble complexes that effectively release the adsorbed metal ions from the nHAP-CGS surface without destroying its structural integrity.^64^ Removal efficiencies in each cycle were used to assess performance decay.The regeneration efficiency (RE, %) was calculated as:

(Equation 13) $$RE\left(\%\right)=\frac{q_{n}}{q_{1}}\times 100\%$$

Three parallel samples were analyzed and averaged.

#### Analysis and characterization

Pb(II), Cu(II) and Cd(II) were measured by flame AAS (GB 7475-87) at 283.3, 324.7 and 228.8 nm; pH was determined by the glass electrode method (GB/T 6920-86).

Surface morphology and elemental composition of CGS and nHAP-CGS before and after adsorption were characterized by SEM–EDS (SIGMA 500+OXFORD Ultim Extreme; 10 μA, 15 kV, Ar protection, 5000–10000×). Phase composition was analyzed by XRD (Bruker D8 Advance, Cu Kα, 40 kV, 40 mA, 2θ = 5–90°, step 0.02°, 0.5 s/step), and patterns were processed using MDI Jade 6.0. Functional groups were examined by FTIR (ALPHA II, Bruker) using KBr pellets (sample <0.074 mm; 400–4000 cm^−1^). Specific surface area, total pore volume and average pore diameter were determined by N_2_ adsorption–desorption (BET, ASAP 2460, Micromeritics) after degassing at 200°C for 360 min (measurement 360 min).

### Quantification and statistical analysis

All experiments were performed in triplicate, and data are presented as mean ± standard deviation. Adsorption isotherm and kinetic models were fitted using nonlinear regression (Origin 2021, Levenberg–Marquardt algorithm). Statistical details are provided in the figure legends and tables.
